# Supplementary material for: Do NSm Virulence Factors in the Bunyavirales Viral Order Originate from Gn Gene Duplication?
Source: Viruses. 2024 Jan 5;16(1):90. doi: 10.3390/v16010090 (PMC10819786; doi:10.3390/v16010090)
Supplement: Supplementary file 1 [file viruses-16-00090-s001.zip › viruses-2760753-supplementary.pdf]

Supporting information

Do NSm virulence factors in the Bunyavirales viral order originate from Gn gene duplication?

Victor Lefebvre[1], Ravy Leon Foun Lin[1], Laura Cole[1], François-Loïc Cosset[2], Marie-Laure Fogeron[1*], and Anja Böckmann[1*]

[1]Molecular Microbiology and Structural Biochemistry (MMSB), UMR 5086 CNRS/Université de Lyon 1, 69367 Lyon, France

[2]Centre International de Recherche en Infectiologie (CIRI), UMR5308 CNRS/Université de Lyon 1/ENS Lyon, U1111 Inserm, 46 allée d'Italie, 69007 Lyon, France

Table S1: pLDDT values for the different Alphafold predictions

Protein	Virus	pLDDT of relaxed models
Nairoviridae		
Gn^{cyto}	CCHFV	model_1: 86<br>model_2: 84<br>model_3: 84<br>model_4: 83<br>model_5: 86
NSm	CCHFV	model_1: 73<br>model_2: 76<br>model_3: 76<br>model_4: 76<br>model_5: 66
NSm	DUGV	model_1: 71<br>model_2: 71<br>model_3: 76<br>model_4: 67<br>model_5: 72
Peribunyaviridae		
Gn^{cyto}	AKAV	model_1: 87<br>model_2: 85<br>model_3: 85<br>model_4: 86<br>model_5: 87
Gn^{cyto}	LACV	model_1: 81<br>model_2: 83<br>model_3: 82<br>model_4: 84<br>model_5: 85
Gn^{cyto}	BUNV	model_1: 85<br>model_2: 82<br>model_3: 86<br>model_4: 86<br>model_5: 86
Gn^{cyto}	TCM	model_1: 85<br>model_2: 84<br>model_3: 88<br>model_4: 87<br>model_5: 88
NSm	AKAV	model_1: 76<br>model_2: 70<br>model_3: 73<br>model_4: 74<br>model_5: 71
NSm	CEV	model_1: 79<br>model_2: 75<br>model_3: 81<br>model_4: 73<br>model_5: 71
NSm	LACV	model_1: 84<br>model_2: 85<br>model_3: 86<br>model_4: 84<br>model_5: 75
NSm	BATV	model_1: 89<br>model_2: 88<br>model_3: 90<br>model_4: 89<br>model_5: 89
NSm	NRIV	model_1: 91<br>model_2: 90<br>model_3: 91<br>model_4: 91
NSm	BUNV	model_1: 82<br>model_2: 78<br>model_3: 77<br>model_4: 79<br>model_5: 75
NSm	UMB	model_1: 78<br>model_2: 66<br>model_3: 73<br>model_4: 77<br>model_5: 68
NSm	WIT	model_1: 77<br>model_2: 78<br>model_3: 81<br>model_4: 77<br>model_5: 79
NSm	TCM	model_1: 85<br>model_2: 87<br>model_3: 90<br>model_4: 87<br>model_5: 83
Phenuiviridae		
Gn^{cyto}	RVFV	model_1: 70<br>model_2: 69<br>model_3: 70<br>model_4: 70<br>model_5: 68
Gn^{cyto}	TOSV	model_1: 66<br>model_2: 64<br>model_3: 70<br>model_4: 66<br>model_5: 63
Gn^{cyto}	AMTV	model_1: 58<br>model_2: 54<br>model_3: 55<br>model_4: 55<br>model_5: 51
Gn^{cyto}	PTPV	model_1: 72<br>model_2: 70<br>model_3: 73<br>model_4: 74<br>model_5: 69
NSm	RVFV	model_1: 49<br>model_2: 45<br>model_3: 49<br>model_4: 42<br>model_5: 30
NSm	TOSV	model_1: 74<br>model_2: 66<br>model_3: 76<br>model_4: 62<br>model_5: 73
NSm	AMTV	model_1: 41<br>model_2: 32<br>model_3: 40<br>model_4: 34<br>model_5: 36
NSm	PTPV	model_1: 78<br>model_2: 78<br>model_3: 78<br>model_4: 78<br>model_5: 78
Tospoviridae		
Gn^{cyto}	TSWV	model_1: 65<br>model_2: 66<br>model_3: 66<br>model_4: 66<br>model_5: 65
NSm	TSWV	model_1: 66<br>model_2: 64<br>model_3: 70<br>model_4: 66<br>model_5: 63

Table S2: **Sequence alignment of the M polyprotein of different CCHFV strains**. The Gn^{cyto} and NSm zinc finger domains are highlighted in green and yellow, respectively, within Gn^{cyto} and full-length NSm in grey. Red type/ asterisk (*), green type/colon (:), and blue type/dot (.) indicate identical amino acid residues, conserved substitution, and semi-conserved substitutions respectively.

```
                            10        20        30        40        50        60        70        80        90       100
                             |         |         |         |         |         |         |         |         |         |
Q8JSZ3              -----MHISLMYAILCLQLCGLGETHGS----HNETRHNKTDTMTTPGDN-PSSEPPVSTALSITLDPSTVTPTTPASGLEGSGEVYTSPPITTGSLPL-
Q70UR1_9VIRU        -----MHISLMYAVFCLQLCGLGKTNGP----HNGTEHNNTHVMTTPDDS-QSPEPPVSTALPVTPDPSTVTPSTPASGLEGSGEVYTSSPITTKGLSL-
A0A345K523_9VIRU    MPINIMHTLLVCFILYLQLLGPGGAHGQ----SNATEHNGTNTTTAPGTS-QSPKPPASTTPSHAPEPSTIKLTTPTSETEGSGET-TPPTNTTQDSPP-
Q0P0I9_9VIRU        MPTNIMHTLLVCFILYLQLLCLGGAHGQ----LNTTEHNGTNNTTAPGAS-QSPKPPMSTTPPHAPESSTIKPTTPISEAEGSGET-TSTPNTTQGLSS-
A0A5K1KE88_9VIRU    MPISIMHISLMCAVLCLQLYGLGGTHGS----HNGTEHNKTDAVTTSSDS-QSSEPPVRTTQPVTPDSSTVTPTTPASVPEGSGEVYTSPLNTTEGPSP-
A0A1V0G0E6_9VIRU    MLRYILYALLASAILHQHLYKVGADTQKPTVRTNTT-HKISPTATANRTTYEQATPSTTPKSSTKP--TTHIATAHLAESDGSGETTLLLPTTASQYTT-
Q8QZF9_9VIRU        MLFHFKFMLLINFILCHLLWGGGGVTGGVETNSSSTQAIQTPPVTSNSTT---PGPSTDTATETASSMTTSTPDTQVTTDNGSGEPSTDPLTTNEATPPL
A0A482LVV7_9VIRU    MLFHFKFMLLINFILCHLLWGGVGVTGGVETNSSSTQAIPTPPVTSNSTT---PGPSTDTATETASSMTTSTPDTQVTTDNGSGEPSTDPLTTNEATPPL
A0A1L3HE22_9VIRU    -MFCLKLVLLINLTLCQLLEGNDRVTSADGINSNTTQTNLGISTILNSTT-PAPGPSTDTTMETVPSTADSMLETQVTTDSGSGEPSTEPTTTGEATSST
                             *    :   *              . *             .   . *.  .        :     :  :  .****       *    .
Prim.cons.          M2F2IMHILL222ILCLQL4GLGG22G3VETNSNTTEHN2TPT2T22ST2YQSP2PPTST233TAP2PST2TPTTP2S2DEGSGE32TSPPTTTE222PL

                           110       120       130       140       150       160       170       180       190       200
                             |         |         |         |         |         |         |         |         |         |
Q8JSZ3              -----------SETTPELPVTTGTDTLSAGDVDPSTQTAGGTSAPTVRTSLPNSPSTPSTPQDTHHPVRNLLSVTSPGPDETSTPSG-TGKESSATSSPH
Q70UR1_9VIRU        -----------PEATSEPPATTSVVTSSASDTDSSTQAAGDTPTPTVRTSLPSSPSTPSTSQGTHYPVRSLLSVTSPKPEETPTPSK-SGKDDLATNSPH
A0A345K523_9VIRU    -----------LETTPERSATTATSTSGTDNMNSTTQTTDNTPTSTVSTSLSSSPSTPSTSQGMHHPVRSLLSVSSSMTATTPTPMS-PGEMSSETSSQH
Q0P0I9_9VIRU        -----------PETTSERPATTAISTSSTDSTNPTTQMTDNTPTLTVSTSPSSSPSTPSTPQGIYHPARSLLSVSSPKTVTTPTPTS-PGEMSSETSSQH
A0A5K1KE88_9VIRU    -----------PESTPESPVAASTGTPSASDVNSSTQAARDTSTPTVRTSPPNSLSTPPTPQETHHPVRNLLSVTSHKPEETTAPSE-SGKESPAANSPH
A0A1V0G0E6_9VIRU    ----------EGQTSLEPTAEETGSNTIPSDTTSTSPITENSESTDTQMNVSDSSSSPLATQGARSPARNLLSVPSVEPGQQTTPKQPTTKGTVTPTTPQ
Q8QZF9_9VIRU        SVTTAQTTETKKHTTLDPSTSTNPDATTPSITILSASNTSLPISS-VHASPSPSSSEEPTPQNAHHLSRSLLSAVSTETGQIASAAT-AAKEITTKNSPP
A0A482LVV7_9VIRU    SVTTAQTKETKKHTTLDPNTSANPDATTPSITILSASNTSLPTSS-VHASPSPSSSEEPTPQNAHHLSRSLLSAVSTETGQTASATT-ATKEITTKNSPP
A0A1L3HE22_9VIRU    TAATVSMQETKKHMTQNTRSTADPDTTTPNSTTSVEPTTIPPTSPIPQATPTPGHGKAPTLPNTHHLSRSLLSAVSTEAGQTTPATS-TTEAASAPSSSS
                                . : :           ..        :  . :     . . . .   :        *.***. *  .   ...   . :     .:
Prim.cons.          SVTTAQT3ETK2ETT2EPPATT32DTTSPSDTNSSTQTT3NTP2PTVRTSPS2SPSTP2TPQGTHHPVRSLLSV2S2E2GQT2TP2SPTGKESSAT2SPH

                           210       220       230       240       250       260       270       280       290       300
                             |         |         |         |         |         |         |         |         |         |
Q8JSZ3              PVSNRPPTPPATAQGPTENDSHNATEHPESLTQSATPGLMTSPTQIVHPQSATPITVQDTHPSPTNRSKRNLKMEIILTLSQGLKKYYGKILRLLQLTLE
Q70UR1_9VIRU        PATSRPTTPPTTAQKPTENNSHNTTEQLESLTHLATLGLMISPTQTVLPQSVTSIAIQDIHNSPTNRSKRNLDMEIILTLSQGLKKYYGKILKLLHLTLE
A0A345K523_9VIRU    PAMSRTPTPHTATQVSTENANRSTSRQSESSAQPATQSPVTSPVQSILLMSATPTTIQNIHPSPTNRSKRNLEMEIILTLSQGLKKYYGKILKLLHLTLE
Q0P0I9_9VIRU        SAMSRIPTPHTATRVSTENTNHSTPRQSESSAQQTTPSPMTSPAQSILLMSAAPTAVQDIHPSPTNRSKRNLETEIILTLSQGLKKYYGKILKLLHLTLE
A0A5K1KE88_9VIRU    PVASRPPTPSTTAQGPTENNSHSTTGQLESLTQSTTLDPMTSLTQTVLPQSVTSIAVQDTHPSPTNRFKRNLEMEIILTLSQGLKKYYGKVLRLLQLTLE
A0A1V0G0E6_9VIRU    PTTSSPTPAPTSATLSTNSSSISTPVQTSSPLQPMAPSHETQHALYTTSTESTLRTLSAERIHPTNRSRRNSEVEIILTLPQGLKKYYSRLLKLLHLTME
Q8QZF9_9VIRU        TAGND-----TMVTSAPNGNIRNTPEQANPTAPEASPKIQTTQNQQTVTPEATPITTITALTDLTNRSKRDSKVQIILTFSQGLKKYYSKILKLLQLTQE
A0A482LVV7_9VIRU    TAGND-----TMVTSAPNGNTRNTPEQANPTAPEASLKIQTTQNQQTVTPEATPITTVTALTDLTNRSKRDSKVQIILTFSQGLKKYYSKILKLLQLTQE
A0A1L3HE22_9VIRU    TAENN-----TVSQPAQDTTTSGVSEQYNPTTTGISPKAQTMQGQQAIVPVTTPVTTTFTPTNPTNRSKRESKVQIILTFPQGLKKYYSKILKLLHLTQE
                    .. .      :    . :    ... : ..     :                :  :        *** :*: . :****:.*******.::*:**:** *
Prim.cons.          PA3SRPPTPPTTAQ33TENNSH2TPEQ3ES22Q3ATP2PMTSPTQQ2LP2SATPIT2QD2HPSPTNRSKRNL22EIILTLSQGLKKYYGKILKLLHLTLE

                           310       320       330       340       350       360       370       380       390       400
                             |         |         |         |         |         |         |         |         |         |
Q8JSZ3              EDTEGLLEWCKRNLGLDCDDTFFQKRIEEFFITGEGHFNEVLQFRTPGTLSTTESTPAGLPTAEPFKSYFAKGFLSIDSGYYSAKCYSGTSNSGLQLINI
Q70UR1_9VIRU        EDTEGLLEWCKRNLGLDCDDTFFQKRIEEFFITGEGHFNEVLQFRTLGTLSTTESTHAGSPTVEPFKSYFAKGFLSIDSGYFSAKCYSRTSNSGLQLINV
A0A345K523_9VIRU    EDTEGLLEWCKRNLGSNCDDDFFQKRIEEFFITGEGYFNEVLQFKTLSTPSSTEPSHARLPTAEPFKSYFAKGFLSIDSGYFSAKCYPRSSTSGLQLINV
Q0P0I9_9VIRU        EDTEGLLEWCKRNLGSNCDDDFFQKRIEEFFMTGEGYFNEVLQFKTLSTLSPTEPSHARLPTAEPFKSYFAKGFLSIDSGYFSAKCYPRSSASGLQLINV
A0A5K1KE88_9VIRU    EDTEGLLEWCKRNLGLDCDDTFFQKRIEEFFITGEGHFNEVLQFRTPGTLSTKESTHAGSPTAEPFKSYFAKGFLSIDSGYFSAKCYSRTSNSGLQLINV
A0A1V0G0E6_9VIRU    EDTEGLQEWCVRVLGQTCDDSYFHKRLAEFFITGTGFFNEVLLFRSPSTPTTIKPSPTGSPTVEPFRSYYAKGSLTIDSGYFSAKCYPRTSNSGLQLINL
Q8QZF9_9VIRU        EDSEGLLEWCTRVLEQACDDDYFNKRIEEFFITGKGYFNDVLQFRLHDALSNTEPAQTTSPTAVPFKSYYAKGSLTFDSGYFSARCYPRASNSGLQLINV
A0A482LVV7_9VIRU    EDSEGLLEWCTRVLEQACDDDYFNKRIEEFFITGKGYFNDVLQFRLHDAISNTEPAQTTSPTAVPFKSYYAKGSLTFDSGYFSARCYPRASNSGLQLINV
A0A1L3HE22_9VIRU    EDSEGLLEWCTRVLQQVCDDTYFNERIEEFFITGKGYFNDVLQFKLHDTPTTTVSALAASSTAMPFRSYYAKGSLTLDSGYFSAKCYPRASNSGMQLINV
                    **:*** *** * *   *** :*::*: ***:** *.**:** *:  .: :   .: :  .*. **:**:*** *::****:**:**. :* **:****:
Prim.cons.          EDTEGLLEWCKRNLGQDCDD2FFQKRIEEFFITGEGYFNEVLQFRT33TLSTTEP3HAGSPTAEPFKSYFAKGFLSIDSGYFSAKCYPRTSNSGLQLINV

                           410       420       430       440       450       460       470       480       490       500
                             |         |         |         |         |         |         |         |         |         |
Q8JSZ3              TRHSTRIVDTPGPKITNLKTINCINLKASIFKEHREVEINVLLPQVAVNLSNCHVVIKSHVCDYSLDIDGAVRLPHIYHEGVFIPGTYKIVIDKKNKLND
Q70UR1_9VIRU        TRHSTRIADTPGPKITNLKTINCMNLKASVFKEHREVEINVLLPQVAVNLSNCHVAIKSHICDYSLDTDGAIRLPQIHHEGTFIPGTYKIVIDKKSRLND
A0A345K523_9VIRU    TQHPARIAETPGPKTTSLKTINCINLRASVFKEHREVEINVLLPQIAVNLSNCHVVINSHVCDYSLDTDGPVRLPRIYHEGTFMPGTYKIVIDRKNKLND
Q0P0I9_9VIRU        TQHPARIAETPGPKTTSLKTINCINLRASVFKEHREIEINVLLPQIAVNLSNCHVVINSHVCDYSLDTDGPVRLPRIYHEGTFIPGTYKIVIDKKNKLND
A0A5K1KE88_9VIRU    TRHSTRIADTPGPKITNLKTISCINLKASVFKEHREVEINVLLPQVAVNLSNCHVTIKSHVCDYSLDIDGTVRLPHIHHDGTFIPGTYKIVIDRKNKLND
A0A1V0G0E6_9VIRU    TQHSVEIANTPGPKHSSLKTINCINIKASTDKEHNEVEINVLLPQVAINLSNCHVSIRSHVCDYSLDTDGKIRLPQISHEGTFIPGTYKIIIDKKNKLND
Q8QZF9_9VIRU        TQHPVKIANTPGPKLSNLKTINCINLKVSTDKEHSELEVNVLLPQVAVNLSNCHVSIKSHVCDYSLDTDGAIKLPRVAHNGVFIPGTYKIVIDKKNNLND
A0A482LVV7_9VIRU    TQHPVKIANTPGPKLSNLKTINCINLKVSTDKEHSELEVNVLLPQVAVNLSNCHVSIKSHVCDYSLDTDGAIKLPRVAHNGVFIPGTYKIVIDKKNNLND
A0A1L3HE22_9VIRU    TQHPMKIANTPGPKTSNLKTMNCINLKVSTDKEHSELEINVLIPQVAVNLSNCLVSIKSHVCDYSLDTDGAIRLPKVAYNGVFMPGTYKIVIDKKNRLND
                    *:*. .*.:***** :.***:.*:*::.*  *** *:*:***:**:*:***** * *.**:****** ** ::**:: ::*.*:******:**:*..***
Prim.cons.          TQHP2RIANTPGPK2TNLKTINCINLKAS2FKEHREVEINVLLPQVAVNLSNCHVSIKSHVCDYSLDTDGAIRLPRI2HEGTFIPGTYKIVIDKKNKLND

                                                       Gn    1        10        20        30        40        50
                                                             |         |         |         |         |         |
                           510       520       530       540       550       560       570       580       590       600
                             |         |         |         |         |         |         |         |         |         |
Q8JSZ3              RCTLFTDCVIKGREVRKGQSVLRQYKTEIRIGKASTGSRRLLSEEPSDDCISRTQLLRTETAEIHGDNYGGPGDKITICNGSTIVDQRLGSELGCYTINR
Q70UR1_9VIRU        RCTLFTNCVIKGREVRKGQSVLRQYKTEIRIGRASTGSRRLLSEESGDDCISRTQLLRTETAEVHGDNYGGPGDKITICNGSTVVDQRLGSELGCYTINR
A0A345K523_9VIRU    RCTLVTNCVIKGREVRKGQSVLRQYKTEIKIGKAPTGSRKLLSEEPGDDCISRTQLLRTETAEIHDDNYGGPGDKITICNGSTIVDQRLGSELGCYTINR
Q0P0I9_9VIRU        RCTLVTNCVIKGREVRKGQSVLRQYKTEIKIGKASTGFRKLLSEEPGDDCISRTQLLRTETAEIHDDNYGGPGDKITICNGSTIVDQRLGSELGCYTINR
A0A5K1KE88_9VIRU    RCTLFTNCVIKGREVRKGQSVLRQYKTEIRIGKASTGSRRLLSDESGDDCISRTQLLRTETAEIHGDNYGGPGDKITICNGSTIVDQRLGSELGCYTINR
A0A1V0G0E6_9VIRU    RCSLITSCVIKGRELRKGQSTLRQYKTEIKIGQTSSGSRRLLSESSASGCISRTQMIKSETAEIHDDIYGGPGEKLTICNGTTVVDQRLGSELGCYTINR
Q8QZF9_9VIRU        RCTLITNCVIKGREVRKGQSALRQYKTEIRIGQTFTGSRRLLAEGGSSDCISRTQLVKTEVAEIHEDSYGGPGEKITICNGSTIVDQRLGSELGCYTINR
A0A482LVV7_9VIRU    RCTLTTNCVIKGREVRKGQSALRQYKTEIRIGQTVTGSRRLLAEGGSSDCISRTQLVKTEVAEIHEDSYGGPGEKITICNGSTIVDQRLGSELGCYTINR
A0A1L3HE22_9VIRU    RCALITNCVIKGREVRKGQSTLRQYKTEIRIGQTLMGSRRLLAEGVSNDCVSRTQLIKTEVAEIHEDSYGGPGEKITICNGSTIVDQRLGSELGCYTINR
                    **:* *.*******:*****.********:**::  * *:**::  ...*:****::::*.**:* * *****:*:*****:*:****************
Prim.cons.          RCTL2TNCVIKGREVRKGQSVLRQYKTEIRIG2ASTGSRRLLSEE22DDCISRTQLLRTETAEIH3DNYGGPGDKITICNGSTIVDQRLGSELGCYTINR

                    60        70        80        90       100       110       120       130       140       150
                     |         |         |         |         |         |         |         |         |         |
                           610       620       630       640       650       660       670       680       690       700
                             |         |         |         |         |         |         |         |         |         |
Q8JSZ3              VRSFKLCENSATGKNCEIDSVPVKCRQGYCLRITQEGRGHVKLSRGSEVVLDACDTSCEIMIPKGTGDILVDCSGGQQHFLKDNLIDLGCPKIPLLGKMA
Q70UR1_9VIRU        VRSFKLCENSATGKSCEIDSIPVKCKQGYCLKITQEGRGHVKLSRGSEVVLDVCDSSCEVMIPKGTGDILVDCSGGQQHFLKDNLVDLGCPKIPLLGKMA
A0A345K523_9VIRU    VKSFKLCENSATGKTCEIDSTPVKCRQGFCLKITQEGRGHVKLSRGSEVVLDACDSNCEVMIPKGTGDILVDCSGGQQHFLKDNLIDLGCPHIPLLGKMA
Q0P0I9_9VIRU        VKSFKLCENSATGKTCEVDSTPVKCRQGFCLKITQEGRGHVKLSRGSEVVLDACDSSCEVMIPKGTGDILVDCSGGQQHFLKDNLIDLGCPHIPLLGKMA
```

```
A0A5K1KE88_9VIRU    VKSFKLCENSATGKSCEIDSIPVKCRQGYCLKITQEGRGHVKLSRGSEVVLDACDSSCEVMIPKGTGDILVDCSGGQQHFLKDNLIDLGCPKIPLLGKMA
A0A1V0G0E6_9VIRU    IKTYKLCENSVKEKTCEIDSVPVKCRQGLCLKITQEGRGHVKLSRGSEIVLDICDSSCELMIPKGTGDILVDCSGGQQHFLQDNLVDLGCPNIPLLGKVA
Q8QZF9_9VIRU        VRSYKLCENSASGKSCEIDSIPVKCKQGFCLKITQEGRGHVKLSRGTEIVLDVCDSSCELMIPKGTGDILVDCSGGQQHFLQNNLIDLGCPNVPLLGKMA
A0A482LVV7_9VIRU    VKSYKLCENSASGKSCEIDSIPVKCKQGFCLKITQEGRGHVKLSRGTEIVLDVCDSSCELMIPKGTGDILVDCSGGQQHFLQNNLIDLGCPNVPLLGKMA
A0A1L3HE22_9VIRU    VKTYKLCENSATEKGCEIDSVPVKCKQGFCLKVTQEGRGHVKLSRGTEIVLDVCDTSCELMIPKGTGDILVDCSGGQQHFLQDNLIDLGCPNVPLLGKMA
                    ::::******.. * **:** ****:** **::*************:*:*** **:.**:*********************::**:*****::*****:*
Prim.cons.          VKSFKLCENSATGKSCEIDSIPVKCRQGFCLKITQEGRGHVKLSRGSEVVLD2CDSSCE2MIPKGTGDILVDCSGGQQHFLKDNLIDLGCPNIPLLGKMA

                    160       170       180       190       200       210       220       230       240       250
                      |         |         |         |         |         |         |         |         |         |
                            710       720       730       740       750       760       770       780       790       800
                              |         |         |         |         |         |         |         |         |         |
Q8JSZ3              IYICRMSNHPKTTMAFLFWFSFGYVITCILCKAIFYLLIIVGTLGKRLKQYRELKPQTCTICETTPVNAIDAEMHDLNCSYNICPYCASRLTSDGLARHV
Q70UR1_9VIRU        IYICRMSNHPKTTMAFLFWFSFGYVITCILCKAIFFLLIIFGTLGKRFKQYRELKPQTCTICETTPVNAIDAEMHDLNCSYNICPYCASRLTSDGLARHV
A0A345K523_9VIRU    IYICRMSNHPKTTMAFLFWFSFGYVITCIFCKALFYSLIIIGTLGKKIKQYRELKPQTCTICETAPVNAIDAEMHDLNCSYNICPYCASRLTSDGLARHV
Q0P0I9_9VIRU        IYICRMSNHPRTTMAFLFWFSFGYVITCISCKALFYSLIIIGTLGKKIKQYRELKPQTCTICETAPVNAIDAEMHDLNCSYNICPYCASRLTSDGLARHV
A0A5K1KE88_9VIRU    IYICRMSNHPKTTMAFLFWFSFGYVITCIVCKAIFYLLIIVGTLGKRFKQYRELKPQTCTICETTPTNAIDAEMHDLNCSYNICPYCASRLTSDGLARHV
A0A1V0G0E6_9VIRU    IYICRMSNHPKTTMAFLFWFSFGYVITCILCKILFYLLIITGTLGKKLKQYRELKPQTCTICESTPVNAIDAEMHDLNCSYNICPYCASRLTSDGLIRHV
Q8QZF9_9VIRU        IYVCRMSNHPKTTMAFLFWFSFGYVITCIACRVLFYLLIAIGTVGKKLKQYRELKPQTCIVCESVPVNAIDAEMHELNCSYNICPYCASRLSTDGLIRHV
A0A482LVV7_9VIRU    IYVCRMSNHPKTTMAFLFWFSFGYVITCIVCRVLFYLLIAIGTVGKKLKQYRELKPQTCIVCESVPVNAIDAEMHELNCSYNICPYCASRLSTDGLIRHV
A0A1L3HE22_9VIRU    IYICRMSNHPKTTMAFLFWFSFGYVITCVVCKVLFYLLIVIGTLGKKLKQYRELKPQTCIVCESIPVNVIDAEMHELNCNYNICPYCASRLTSDGLVRHV
                    **:*******:*****************: *: :*: **  **:**::*********** :**: *.*.******:***.***********::*** ***
Prim.cons.          IYICRMSNHPKTTMAFLFWFSFGYVITCI2CKALFYLLIIIGTLGKKLKQYRELKPQTCTICETTPVNAIDAEMHDLNCSYNICPYCASRLTSDGLARHV

                    260       270       280       290       300       310        NSm  1        10        20        30
                      |         |         |         |         |         |               |         |         |
                            810       820       830       840       850       860       870       880       890       900
                              |         |         |         |         |         |         |         |         |         |
Q8JSZ3              IQCPKRKEKVEETELYLNLERIPWVVRKLLQVSESTGVALKRSSWLIVLLVLFTVSLSPVQSAPIGQGKTIEAYRAREGYTSICLFVLGSILFIVSCLMK
Q70UR1_9VIRU        TQCPRRKEKVEETELYLNLERIPWVVRKLLQVSESTGTVLKRSSWLIVLLVLFTVSLSPVQSAPIGHGKTIETYRVREEYTSICLFVLGSILFIVSFLMK
A0A345K523_9VIRU    KQCPKRKEKVEETELYLNLERIPWVVRKLLQVSESTGVALKRSSWLIVLLVLLTVSLSPVQSAPVGHGKTIETYQTREGFTSICLFMLGSILFIVSCLVK
Q0P0I9_9VIRU        TQCPKRKEKVEETELYLNLERIPWIVRKLLQVSESTGVALKRSSWLIVLLVLLTVSLSPVQSAPVGHGKTIEIYQTREGFASICLFMLGSILFIVSCLVK
A0A5K1KE88_9VIRU    THCPKRKEKVEETELYLNLERIPWVVRKLLQVSESTGVALKRSSWLIVLLVLFAISLSPVQSAPISHGKTIEIYQVREEYTSICLFVLGSILFIVSCLMK
A0A1V0G0E6_9VIRU    NLCPKRKEKVEETELYLNLERIPWFVRKLLQVSESTGVALKRSSWLLILVILLTVSMSPVQSAPINQQRAVEAYQAREGYTGICLFILGSVLFMTSWLTK
Q8QZF9_9VIRU        TQCPKRREKVEETELFLNLERIPWLVRKLLQVSESTGMALKRSCWMITLLILLLVSISPVQSAPVGKERAIETYQARETYTGICLFVLGSVLFAVSWLTK
A0A482LVV7_9VIRU    TQCPKRREKVEETELFLNLERIPWLVRKLLQVSESTGMALKRSCWMITLLILLLVSMSPVQSAPVGKERAIETYQAREAYTGICLFVLGSVLFAVSWLTK
A0A1L3HE22_9VIRU    TQCPKRKEKVEETELYLNLERIPWLVRRLLQVSESTGMALKRSCWMTTLLILLVVSLSPVQSAPVGQEKAIEVYQVRESYTSMCLFVLGSVLFAVSWLVK
                      **:*:********:********.**:*********  .****.*:   *::*:  :*:*******:.:  :::* *:.** ::.:***:***:**  .* * *
Prim.cons.          TQCPKRKEKVEETELYLNLERIPWVVRKLLQVSESTGVALKRSSWLIVLLVLLTVSLSPVQSAPVGHGKTIETYQAREGYTSICLFVLGSILFIVS2L3K

                            40        50        60        70        80        90        100       110       120       130
                              |         |         |         |         |         |         |         |         |         |
                            910       920       930       940       950       960       970       980       990      1000
                              |         |         |         |         |         |         |         |         |         |
Q8JSZ3              GLVDSVGNSFFPGLSICKTCSISSINGFEIESHKCYCSLFCCPYCRHCSTDKEIHKLHLSICKKRKKGSNVMLAVCKLMCFRATMEVSNRALFIRSIINT
Q70UR1_9VIRU        GLVDGVGNIFFPGLSVCKTCSIGSINGFEIESHKCYCSLFCCPYCRHCSADGEIHQLHLSICKKRKTGSNVMLAVCKRMCFRATMEVSNKALFIRSIINT
A0A345K523_9VIRU    GLVDSVSDSFFPGLSVCKTCSIGSINGFEIESHKCYCSLFCCPYCRHCSADREIHQLHLSICKKRKTGSNVMLAVCKRMCFRATIEASRRALLIRSIINT
Q0P0I9_9VIRU        GLVDSVSDSFFPGLSVCKTCSIGSVNGFEIESHKCYCSLFCCPYCRHCSADREIHQLHLSICKKRKTGSNVMLAVCKRMCFRATIEASRRALLIRSIINT
A0A5K1KE88_9VIRU    GLVNSVGNIFFPGLSICKTCSIGSINGFEIESHKCYCSLLCCPYCRHCSADREIHQLHLSICKKRKTGSNVMLAVCKRMCFKATMEVSNKALFIRSIINT
A0A1V0G0E6_9VIRU    GLIDSIGDSFFPGLFVCKTCSIGSINGFEIESHRCYCSLLCCPYCRTCSADKDSHQLHLSICKKKKAGSNVMLAVCKRMCFKATIEASNRVLLIRSIINT
Q8QZF9_9VIRU        ALIDGIGNSFFPGLSVCKTCSIGSINGFEIESHKCYCSLFCCPYCRACSSDKNTHRMHLNVCKKRKMGSNVMLAVCKRMCFRATIEASNKALLIRSIINS
A0A482LVV7_9VIRU    ALIDGIGNSFFPGLSVCKTCSIGSINGFEIESHKCYCSLFCCPYCRACSSDKNTHRMHLNVCKKRKMGSNVMLAVCKRMCFRATIEASNKALLIRSIINS
A0A1L3HE22_9VIRU    GLIDGIGNSFFPGLSVCKTCSIGSINGFEIESHKCYCSLFCCPYCRACSSDKITHRMHLNICKRRKVGSNVMLAVCKRMCFKATVEASNSALLIRSIINS
                    .*::.:.: ***** :******.*:********:*****:****** **:*   *::**.:**::* ********** ***:**:*.*.  .*:******:
Prim.cons.          GLVDSVGNSFFPGLSVCKTCSIGSINGFEIESHKCYCSLFCCPYCRHCSADKEIHQLHLSICKKRKTGSNVMLAVCKRMCFRATIEASN2ALLIRSIINT

                           140       150
                             |         |
                           1010      1020      1030      1040      1050      1060      1070      1080      1090      1100
                              |         |         |         |         |         |         |         |         |         |
Q8JSZ3              TFVLCILILAVCVV-STSAVEMENLPAGTWEREEDLTNFCHQECQVTETECLCPYEALVLRKPLFLDSTAKGMKNLLNSTSLETSLSIEAPWGAINVQST
Q70UR1_9VIRU        TFVMCILKGEFAAVKSIHPIEMESLPAGTWEREEDLTNFCHQECQVTETECLCPYEALVLRRPLFLDSIVKGMKNLLNSTSLETSLSIEAPWGAINVQST
A0A345K523_9VIRU    TFVICILTLTICVV-STSAVEMENLPAGTWEREEDLTNFCHQECQVTETECLCPYEALVLRKPLFLDSIVKGMKNLLNSTSLETSLSIEAPWGAINVQST
Q0P0I9_9VIRU        TFVICILTLTICVV-STSAVEMENLPAGTWEREEDLTNFCHQECQVTETECLCPYEALVLRKPLFLDSIVKGMKNLLNSTSLETSLSIEAPWGAINVQST
A0A5K1KE88_9VIRU    TFVVCILILAVCVV-STSAVEMENLPAGTWEREEDLTNFCHQECQVTETECLCPYEALVLRRPLFLDSIVKGMKNLLNSTSLETSLSIEAPWGAINVQST
A0A1V0G0E6_9VIRU    TFIICMLILVVCVI-STSAVEMEDLPAGTWEREEDLTNFCHQECQVTETECLCPYEAFVLRKPLFLSSIVKGKKNLLNSASLETSLSIEAPWGAINAQST
Q8QZF9_9VIRU        TFVICILILTICVV-STSAVDMENLPAGIWEKEEDLTNFCHQECQVTETECLCPYEAMVLRKPLFLDNIAKGMKSLLNSTSLETSLSIEAPWGAINVQST
A0A482LVV7_9VIRU    TFVICILILTICVV-STSAVDMENLPAGIWEKEEDLTNFCHQECQVTETECLCPYEAMVLRKPLFLDSIAKGMKSLLNSTSLETSLSIEAPWGAINVQST
A0A1L3HE22_9VIRU    TFVICVLILVICVV-STSAVDMENLPAGIWEREEDLTNFCHQECQVTETECLCPYEALILRKPLYLDSIAKGMKSLLNSTNLETSLSIEAPWGAINVQST
                    **::*:*    ...: *   .::**.**** **:************************::**:**:*..  .** *.****:.***************.***
Prim.cons.          TFVICILILTICVVKSTSAVEMENLPAGTWEREEDLTNFCHQECQVTETECLCPYEALVLRKPLFLDSIVKGMKNLLNSTSLETSLSIEAPWGAINVQST

                           1110      1120      1130      1140      1150      1160      1170      1180      1190      1200
                              |         |         |         |         |         |         |         |         |         |
Q8JSZ3              YKPTVSTANIALSWSSVEHRGNKILVSGRSESIMKLEERTGISWDLGVEDASESKLLTVSVMDLSQMYSPVFEYLSGDRQVGEWPKATCTGDCPERCGCT
Q70UR1_9VIRU        YKPTVSTANIALSWSSVEHRGNKVLVSGRSESIMKLEERTGISWDLGVEDASESKLLTVSVMDLSQMYSPVFEYLSGDRQVEEWPKATCTGDCPERCGCT
A0A345K523_9VIRU    FKPTVSTANIALSWSSVEHRGNKILVTGRSESIMKLEERTGVSWDLGVEDASESKLLTVSIMDLSQMYSPVFEYLSGDRQVEEWPKATCTGDCPQRCGCT
Q0P0I9_9VIRU        FKPAVSTANIALSWSSVEHRGNKILVTGRSESIMKLEERTGVSWDLGVEDASESKLLTVSIMDLSQMYSPVFEYLSGDRQVEEWPKATCTGDCPERCGCT
A0A5K1KE88_9VIRU    YKPTVSAANIALSWSSVEHRGNKILVSGRSESIMKLEERTGISWDLGVEDASESKLLTVSVMDLSQMYSPVFEYLSGDRQVEEWPKATCTGDCPERCGCT
A0A1V0G0E6_9VIRU    FKPTISTANIALSWSSVEHRGNKVLVSGRSESIMKLEERTGISWNLGVEDASESRMLTVSVMDLSQMYLPVFEYLSGDRQVEEWPKATCTGDCPERCGCT
Q8QZF9_9VIRU        FKPTVSAANIALSWSSVEHRGNKILVSGRSESIMKLEERTGVSWNLGVEDASESKTLTVSVMDLSQMYSPVFEYLSGDRQVEEWPKATCTGDCPERCGCT
A0A482LVV7_9VIRU    FKPTVSAANIALSWSSVEHRGNKILVSGRSESIMKLEERTGVSWNLGVEDASESKTLTVSVMDLSQMYSPVFEYLSGDRQVEEWPKATCTGDCPERCGCT
A0A1L3HE22_9VIRU    FKPTVSAANIALSWSSVEHRGNKILVSGRSESIMKLEERTGISWSLGVEDASESKTLTVSVMDLSQMYSPVFEYLSGDRQVEEWPKATCTGDCPERCGCT
                    :**::*:****************:**:**************:**.*********:  ****:******* ************ ************:*****
Prim.cons.          FKPTVSTANIALSWSSVEHRGNKILVSGRSESIMKLEERTGISWDLGVEDASESKLLTVSVMDLSQMYSPVFEYLSGDRQVEEWPKATCTGDCPERCGCT

                           1210      1220      1230      1240      1250      1260      1270      1280      1290      1300
                              |         |         |         |         |         |         |         |         |         |
Q8JSZ3              SSTCLHKEWPHSRNWRCNPTWCWGVGTGCTCCGLDVKDLFTDYMFVKWKVEYIKTEAIVCVELTSQERQCSLIEAGTRFNLGPVTITLSEPRNIQQKLPP
Q70UR1_9VIRU        SSTCLHKEWPHSRNWRCNPTWCWGVGTGCTCCGLDVKDLFTDYMFVKWKVEYIKTEAIVCVELTSQERQCSLIEAGTRFNLGSVTITLSEPRNIQQKLPP
A0A345K523_9VIRU    SSTCLHKEWPHSRNWRCNPTWCWGVGTGCTCCGVDVKDLFTDHMFVKWKVEYIKTEAIVCVELTSQERQCSLIEAGTRFNLGPVTITLSEPRNIQQKLPP
Q0P0I9_9VIRU        SSTCLHKEWPHSRNWRCNPTWCWGVGTGCTCCGVDVKDLFTDHMFVKWKVEYIKTEAIVCVELTSQERQCSLIEAGTRFNLGPVTITLSEPRNIQQKLPP
A0A5K1KE88_9VIRU    SSTCLHKEWPHSRNWRCNPTWCWGVGTGCTCCGLDVKDLFTDYMFVKWKVEYIKTEAIVCVELISQERQCSLIEAGTRFNLGPVTITLSEPRNIQQKLPP
A0A1V0G0E6_9VIRU    SSTCLHKEWPHSRNWRCNPTWCWGVGTGCTCCGLDVKDLFTDYMFVKWKVEYIKTEAIVCVELTSQERQCSLIEAGTRFNLGPVTITLSEPRNIQQKLPS
Q8QZF9_9VIRU        SSTCLHKEWPHSRNWRCNPTWCWGVGTGCTCCGLDVKDFFTDYMLVKWKVEYIKTEAIVCVELTSQERQCSLIEAGTRFNLGPVTITLSEPRNVQQRLPP
A0A482LVV7_9VIRU    SSTCLHKEWPHSRNWRCNPTWCWGVGTGCTCCGLDVKDFFTDYMLVKWKVEYIKTEAIVCVELTSQERQCSLIEAGTRFNLGPVTITLSEPRNVQQRLPP
A0A1L3HE22_9VIRU    SSTCLHKEWPHSRNWRCNPTWCWGVGTGCTCCGLDVRDLFTDYMMVKWKVEYVKTEAIMCVELTSQERQCSLIEAGTRFNLGPVTITLSEPRNVQQRLPS
                    *********************************:**:*:***:*:*******:*****:**** *****************.**********:**:**.
Prim.cons.          SSTCLHKEWPHSRNWRCNPTWCWGVGTGCTCCGLDVKDLFTDYMFVKWKVEYIKTEAIVCVELTSQERQCSLIEAGTRFNLGPVTITLSEPRNIQQKLPP

                           1310      1320      1330      1340      1350      1360      1370      1380      1390      1400
```

```
                         |         |         |         |         |         |         |         |         |         |
Q8JSZ3              EIITLHPRIEEGFFDLMHVQKVLSASTVCKLQSCTHGVPGDLQVYHIGNLLKGDKVNGHLIHKIEPHFNTSWMSWDGCDLDYYCNMGDWPSCTYTGVTQH
Q70UR1_9VIRU        EIITLHPKIEEGFFDLMHVQKVLSASTVCKLQSCTHGVPGDLQVYHIGNLLKGDRVNGHLIHKIEQHFNTSWMSWDGCDLDYYCNMGDWPSCTYTGVTQH
A0A345K523_9VIRU    EIITLHPKIEEGFFDLMHVQKVLSASTVCKLQSCTHGIPGDLQVYHIGNLLKGDRVNGHLIHKIEPYFNTSWMSWDGCDLDYYCNMGDWPSCTYTGVTQH
Q0P0I9_9VIRU        EIITLHPKIEEGFFDLMHVQKVLSASTVCKLQSCTHGIPGDLQVYHIGNLLKGDRVNGHLIHKIESHFNTSWMSWDGCDLDYYCNMGDWPSCTYTGVTQH
A0A5K1KE88_9VIRU    EIITLHPRIEEGFFDLMHVQKVLSASTVCKLQSCTHGVPGDLQVYHIGNLLKGDKVNGHLIHKIEPHFNTSWMSWDGCDLDYYCNMGDWPSCTYTGVTQH
A0A1V0G0E6_9VIRU    EIVTLHPKIEEGFFDLMHVQKVLSASTVCKLQSCTHGVAGDLQVYHVGNLLKGDKINGHSIHKIEPHLNTSWMSWDGCDLDYYCNMGDWPSCTYTGVTQH
Q8QZF9_9VIRU        EIVTLHPKIEEGFFDLMHVQKILSASTVCKLQSCTHGVPGDFQVYHVGNLLRGDKVNGHSIHKIEPHFNTSWMSWDGCDLDYFCNMGDWPSCTYTGVTQH
A0A482LVV7_9VIRU    EIVTLHPKIEEGFFDLMHVQKILSASTVCKLQSCTHGVPGDFQVYHVGNLLRGDKVNGHSIHKIEPHFNTSWMSWDGCDLDYFCNMGDWPSCTYTGVTQH
A0A1L3HE22_9VIRU    EIVTLHPKIEEGFFDLMHVQKVLFCKHSLQATSCTHGVPGDLQVYHVGNLLKGDKVNGHSIHKIESHFNTSWMSWDGCDLDYFCNMGDWPSCTYTGVTQH
                    **:****:*************:* ..   :  *****:.**:****:****:**::*** *****  ::**************:****************

Prim.cons.          EIITLHPKIEEGFFDLMHVQKVLSASTVCKLQSCTHGVPGDLQVYHIGNLLKGDKVNGHLIHKIEPHFNTSWMSWDGCDLDYYCNMGDWPSCTYTGVTQH

                          1410      1420      1430      1440      1450      1460      1470      1480      1490      1500
                             |         |         |         |         |         |         |         |         |         |
Q8JSZ3              NHASFVNLLNIETDYTKNFHFHSKRVTAHGDTPQLDLKARPTYGAGEITVLVEVADMELHTKKIEISGLKFASLACTGCYACSSGISCKVRIHVDEPDEL
Q70UR1_9VIRU        NHASFVNLLNIETDYTKTFHFHSKRVTAHGDTPQLDLKARPTYGAGEITVLVEVADMELHTKKIEISGLKFAGLTCTGCYACSSGISCKVRIHVDEPDEL
A0A345K523_9VIRU    NHAAFVNLLNIETDYTKTFHFHSKRVTAHGDTPQLDLKARPTYGAGEITVLVEVADMELHTKKVEISGLKFASLACTGCYACSSGISCKVRIHVDEPDEL
Q0P0I9_9VIRU        NHAAFVNLLNIETDYTKTFHFHSKRVTAHGDTPQLDLKARPTYGAGEITVLVEVADMELHTKKVEISGLKFASLACTGCYACSSGISCKVRIHVDEPDEL
A0A5K1KE88_9VIRU    NHASFVNLLNIETDYTKSFHFHSKRVTAHGDTPQLDLKARPNYGAGEITVLVEVADMELHTKKIEISGLKFASLTCTGCYACSSGISCKVRIHVDEPDEL
A0A1V0G0E6_9VIRU    NHAAFVNLLNIETDYTKSFHFHSKRVTAHGDTPQLDLKARPMYGAGEITVLVEVADMELHSKNIEISGLKFASLTCSGCYACSSGISCKVRIHVDEPDEL
Q8QZF9_9VIRU        NRAAFINMLNIETDYTKTFHFHSKRVTAHGDTPQLDLKARPAYGAGEVTVLVEVADLELHTKKLEVSGLKIASLTCSGCYACSSGISCKVRIHVNEPDEF
A0A482LVV7_9VIRU    NRAAFINMLNIETDYTKTFHFHSKRVTAHGDTPQLDLKARPAYGAGEVTVLVEVADLELHTKKLEVSGLKIASLTCSGCYACSSGISCKVRIHVNEPDEF
A0A1L3HE22_9VIRU    NRAAFVNMLNIETDYTKTFHFHSKRVTAHGDTPQLDLKARPTYGAGEVTVLVEVADLELHTKKIEVSGLKFASLTCSGCYACSSGISCKVRIHVNEPDEF
                    *:*:*:*:*********.*********************** *****:********:***:*::*:****:*.*:*:*****************:****:

Prim.cons.          NHAAFVNLLNIETDYTKTFHFHSKRVTAHGDTPQLDLKARPTYGAGEITVLVEVADMELHTKKIEISGLKFASLTCTGCYACSSGISCKVRIHVDEPDEL

                          1510      1520      1530      1540      1550      1560      1570      1580      1590      1600
                             |         |         |         |         |         |         |         |         |         |
Q8JSZ3              TVHVKSDDPDVVAASSSLMARKLEFGTDSTFKAFSAMPKTSLCFYIVEREHCKSCSEEDTKKCVNTKLEQPQSILIEHKGTIIGKQNSTCTAKASCWLES
Q70UR1_9VIRU        TVHVKSDDPDVVAASSSLMARKLEFGTDSTFKAFSAMPKTSLCFYIVEREYCKSCNKEDTQKCVNTKLEQPQSILIEHKGTIIGKQNNTCTAKASCWLES
A0A345K523_9VIRU    TVHVKSSDPDVVAAGTSLMARKLEFGTDSTFKAFSAMPKTSLCFYIVEKEYCKSCSEDDTKKCVDTKLEQPQSILIEHRGTIIGKQNDTCTAKASCWLES
Q0P0I9_9VIRU        TVHVKSSDPDVVAASTSLMARKLEFGTDSTFKAFSAMPKTSLCFYIVEREYCKSCSEDDTQKCVDTRLEQPQSILIEHKGTIIGKQNDTCTAKASCWLES
A0A5K1KE88_9VIRU    TVHVKSDDPDVVAASSSLMARKLEFGTDSTFKAFSAMPKTSLCFYIVEREYCKSCSKEDTQKCVNTKLEQPQSILIEHKGTIIGKQNNTCTSKTSCWLES
A0A1V0G0E6_9VIRU    AVHVKSDDLDVVAASSSLMARKIEFGTDSTFKAFSSMPKESLCFYIVERDFCKSCAAEDTQKCVKAKLEQPQSILIEHKGTIIGKQNDTCSTKTSCWLES
Q8QZF9_9VIRU        TVHVRSSDPDVVAAGSSLMARKIEFGADSTFKAFSSMPKDSLCFYIVEKDYCSSCTDDDTQKCVNTKLDHPQSILIEHKGTIIGKQNDTCSTKTSCWLES
A0A482LVV7_9VIRU    TVHVRSSDPDVVAAGSSLMARKIEFGADSTFKAFSSMPKDSLCFYIVEKDYCSSCTDDDTQKCVNTKLDHPQSILIEHKGTIIGKQNDTCSTKTSCWLES
A0A1L3HE22_9VIRU    TVHVKSNDPDVVAAGSSLMARKIELGADSTFKAFSSMPKNSLCFYIVERDYCSSCTDDDTQKCVNTKLEHPQSILIEHKGTIIGKQNDTCPTKTSCWLES
                    :***:*.* *****.:******:*:*:********:***  ********::.*.**   :**:***.::*::********:********.**.:*:******

Prim.cons.          TVHVKS2DPDVVAASSSLMARKLEFGTDSTFKAFSAMPKTSLCFYIVEREYCKSCS2DDTQKCVNTKLEQPQSILIEHKGTIIGKQNDTCT2KTSCWLES

                          1610      1620      1630      1640      1650      1660      1670      1680      1690      1700
                             |         |         |         |         |         |         |         |         |         |
Q8JSZ3              VKSFFYGLKNMLSGIFGNVFMGIFLFLAPFILLILFFMFGWRILFCFKCCRRTRGLFKYRHLKDDEETGYRRIIEKLNNKK-GKNKLLDGERLADRRIAE
Q70UR1_9VIRU        VKSFFYGLKNMLGGIFGNVFIGIFTFLTPFILLILFFMFGWRILFCFKCCRRTRGLFKYRHLKDDEETGYRKIIERLNNKK-GKNRLLDGERLADRKIAE
A0A345K523_9VIRU    VKSFFYGLKNMLGSVFGNFFIGILLFLAPFVLLVLFFMFGWKILFCFKCCRRTRGLFKYRHLKDDEETGYRRIIERLNNKK-GKNRLLDGERLADRKIAE
Q0P0I9_9VIRU        VKSFFYGLKNMLGSVFGNLFIGILLFLAPFVLLVLFFMFGWKILFCFKCCRRTRGLFKYRHLKDDEETGYRRIIERLNSKK-GKNRLLDGDRLADRKIAE
A0A5K1KE88_9VIRU    VKSFFYGLKNMLSSIFGNVFVGIFLFLAPFILLILFFMFGWRILFCFKCCRRTRGLFRYKHLEDDEETGYRRIIERLNNKK-GKNKLLDGERLADRRIAE
A0A1V0G0E6_9VIRU    FKSFFYGLKNMLSGVFGSAIMGILLFLAPFILLILFFFFGWRILFCLRCCRRTRGLFKYKHIKDDEEAGYKKIIEKLNNKKGGTSKLLDGERLADRKIAE
Q8QZF9_9VIRU        VKGFFYGLKNMLSGIFGNVFLGIFLFLAPFVLLIFFFFFGWRLLFCLKCCRRTRGLLKYKHLKDEEEAGYKKIIERLNGKK-GKSQLFDGERLADRKIAE
A0A482LVV7_9VIRU    VKGFFYGLKNMLSGIFGNVFLGILLFLAPFVLLIFFFFFGWRLLFCLKCCRRTRGLLKYKHLKDEEEAGYKKIIERLNGKK-GKSQLFDGERLADRKIAE
A0A1L3HE22_9VIRU    VKSFFYGLKNMLGGIFGNVFLGIFLFLAPFALLILFFFFGWRLLFCLKCCRRTRGLLKYKQLKDEEEAGYKKIIERLNDKK-GKSRLFDGERLADRKIAE
                    .*.*********..:**.  ::**:  **:**  **::**:***::***::********::*::::*:**:**::***:**.** *..:*:**:*****:***

Prim.cons.          VKSFFYGLKNMLSGIFGNVF2GIFLFLAPF2LLILFFMFGWRILFCFKCCRRTRGLFKYKHLKDDEETGYRKIIERLNNKKGGKNRLLDGERLADRKIAE


Q8JSZ3              LFSTKTHIG
Q70UR1_9VIRU        LFSTKTHIG
A0A345K523_9VIRU    LFSTKTHIG
Q0P0I9_9VIRU        LFSTKTHIG
A0A5K1KE88_9VIRU    LFSTKTHIG
A0A1V0G0E6_9VIRU    LFSTKAHIG
Q8QZF9_9VIRU        LFSTKIHIG
A0A482LVV7_9VIRU    LFSTKIHIG
A0A1L3HE22_9VIRU    LFSTKTHIG
                    ***** ***

Prim.cons.          LFSTKTHIG
```

Table S3: Sequence alignments and numbering of CCHFV and DUGV M polyproteins. Aligned NSm sequences shown in Figure 3b are highlighted in yellow. Red type/ asterisk (*), green type/colon (:), and blue type/dot (.) indicate identical amino acid residues, conserved substitution, and semi-conserved substitutions respectively.

```
                      10        20        30        40        50        60        70        80        90       100
                       |         |         |         |         |         |         |         |         |         |
Q02004_DUGV   MSKRVLIIAVVVYL-------------------------------------------------------------VF-----------------------
Q8JSZ3_CCHFV  MHISLMYAILCLQLCGLGETHGSHNETRHNKTDTMTTPGDNPSSEPPVSTALSITLDPSTVTPTTPASGLEGSGEVYTSPPITTGSLPLSETTPELPVTT
              *   ::   : : *                                                             *:
Prim.cons.    M222222222222LCGLGETHGSHNETRHNKTDTMTTPGDNPSSEPPVSTALSITLDPSTVTPTTPASGLEGSGEV2TSPPITTGSLPLSETTPELPVTT

                     110       120       130       140       150       160       170       180       190       200
                       |         |         |         |         |         |         |         |         |         |
Q02004_DUGV   ------------TTQNQITGNHTTINSSSPSTTEASSTP------------TVSRTPQTTTTSTAVSTTITATTTP------------TASWTTQSQYFN
Q8JSZ3_CCHFV  GTDTLSAGDVDPSTQTAGGTSAPTVRTSLPNSPSTPSTPQDTHHPVRNLLSVTSPGPDETSTPSGTGKESSATSSPHPVSNRPPTPPATAQGPTENDSHN
                          :**.    . .*:.:* *.:..:.***            ..*  *: *:*.:....  :**::*            **. .*:.: .*
Prim.cons.    GTDTLSAGDVDP2TQ22222222T222S2P222222STPQDTHHPVRNLLS22S22P22T2T222222222AT22PHPVSNRPPTPPATA222T22222N

                     210       220       230       240       250       260       270       280       290       300
                       |         |         |         |         |         |         |         |         |         |
Q02004_DUGV   KTTQH-------------------HWREETMIS---RNPTVLDRQSRASSVRELLNT---------KFLMLLGFIPKGEVNHLENACNRE-GKNCTELIL
Q8JSZ3_CCHFV  ATEHPESLTQSATPGLMTSPTQIVHPQSATPITVQDTHPSPTNRSKRNLKMEIILTLSQGLKKYYGKILRLLQLTLEEDTEGLLEWCKRNLGLDCDDTFF
               * :                    * :. * *:    :*:  :*..*  .:. :*.          *:* ** :  : :.: * : *:*: * :* : ::
Prim.cons.    2T222ESLTQSATPGLMTSPTQIVH2222T2I2VQD22P2222R22R2222222L22SQGLKKYYGK2L2LL2222222222L222C2R2LG22C22222

                     310       320       330       340       350       360       370       380       390       400
                       |         |         |         |         |         |         |         |         |         |
Q02004_DUGV   KERIARFFSETEKESCYNTYLEKHLRSVSPEVSLTPYRVLGLREDILLKE-IDRRIIRFETDSQRVTCLSAS----LLKPDVFIREQRIDAKPSNGPKIV
Q8JSZ3_CCHFV  QKRIEEFF--ITGEGHFNEVLQFRTPGTLSTTESTP---AGLPTAEPFKSYFAKGFLSIDSGYYSAKCYSGTSNSGLQLINITRHSTRIVDTP--GPKIT
              ::** .**     *. :*  *: :  .. . .. **    **     :*. : : :: :::.   ..* *.:    *   ::  :. **  .*  ****.
Prim.cons.    22RI22FFSE222E222N22L2222222222222TPYRV2GL222222K2Y2222222222222222C2S22SNSGL2222222222RI222PSNGPKI2

                     410       420       430       440       450       460       470       480       490       500
                       |         |         |         |         |         |         |         |         |         |
Q02004_DUGV   PVDSVACMNLEANVDVRSNKLVIQSLMTTVKISLKNCKVVVNSRQCIHQQTGSGVIKVPKFEKQQGGTWSSYIAGVYTATIDLLDENNQNCKLFTECIVK
Q8JSZ3_CCHFV  NLKTINCINLKASIFKEHREVEINVLLPQVAVNLSNCHVVIKSHVCDYSLDIDGAVRLPHIYHE-----GVFIPGTYKIVIDKKNKLNDRCTLFTDCVIK
               :.:: *:**:*.:  . .:: *: *:. * :.*.**:**::*: * :.   .*.:::*:: ::     . :*.*.*. .**  :: *:.*.***:*::*
Prim.cons.    222222C2NL2A2222222222I22L222V222L2NC2VV22S22C2222222G2222P22222QGGTW222I2G2Y222ID22222N22C2LFT2C22K

                     510       520       530       540       550       560       570       580       590       600
                       |         |         |         |         |         |         |         |         |         |
Q02004_DUGV   GRELVKGQSELKSFNIEVLLPRVMKTRRKLLAVTDGSTECNSGTQLIEGKSIEVHKQDIGGPGKKLTICNGTSVLDVPLDEGHGCYTINVITSKRACRPK
Q8JSZ3_CCHFV  GREVRKGQSVLRQYKTEIRIGKASTGSRRLLS-EEPSDDCISRTQLLRTETAEIHGDNYGGPGDKITICNGSTIVDQRLGSELGCYTINRVRSFKLCENS
              ***: **** *:.:: *: : :. .  *:**:  : * :* * ***:. :: *:* :: ****.*:*****::::*  *..  ****** : * : *. .
Prim.cons.    GRE22KGQS2L22222E2222222222R2LL2V222S22C2S2TQL222222E2H2222GGPG2K2TICNG2222D22L2222GCYTIN222S222C222

                     610       620       630       640       650       660       670       680       690       700
                       |         |         |         |         |         |         |         |         |         |
Q02004_DUGV   NSKLQCSIDKELKPCDSGKCLSISQKGAGHIKVSRGKTILITECKEHCQIPVPTGKGDIMVDCSGGRQHYLEVNIVDIHCPNTKFLGGIMLYFCRMSSRP
Q8JSZ3_CCHFV  ATGKNCEIDSVPVKCRQGYCLRITQEGRGHVKLSRGSEVVLDACDTSCEIMIPKGTGDILVDCSGGQQHFLKDNLIDLGCPKIPLLGKMAIYICRMSNHP
               :  :*.**.    *  .* ** *:*:* **:*:***. :::  *.  *:* :*.*.***:******:**:*: *::*: **:  :** : :*:****.:*
Prim.cons.    22222C2ID22222C22G2CL2I2Q2G2GH2K2SRG2222222C222C2I22P2G2GDI2VDCSGG2QH2L22N22D22CP2222LG2222Y2CRMS22P

                     710       720       730       740       750       760       770       780       790       800
                       |         |         |         |         |         |         |         |         |         |
Q02004_DUGV   TVALLLGIWIGCGYILTCIFSFLLYHLILFFANCIKQCRKKGERLGEICVKCEQQTVNLMDQELHDLNCNFNLCPYCCNRMSDEGMSRHVGKCPKRLERL
Q8JSZ3_CCHFV  KTTMAFLFWFSFGYVITCILCKAIFYLLIIVGTLGKRLKQYRELKPQTCTICETTPVNAIDAEMHDLNCSYNICPYCASRLTSDGLARHVIQCPKRKEKV
              ..:: : :*:. **::***:.  :::*:::...  *: ::  *   : *. **  .** :* *:*****.:*:****..*::.:*::*** :**** *::
Prim.cons.    22222222W222GY22TCI2222222L22222222K222222E22222C22CE222VN22D2E2HDLNC22N2CPYC22R2222G22RHV22CPKR2E22

                     810       820       830       840       850       860       870       880       890       900
                       |         |         |         |         |         |         |         |         |         |
Q02004_DUGV   NEIELYLTTSEC-LCLSVCYQLLISVGIFLKRTTWLVVLLVLLGLAISPVQGAP-----TEVSNVKQDGDYSICYFIFGC-LVTAALLLKVKRTNSNGIV
Q8JSZ3_CCHFV  EETELYLNLERIPWVVRKLLQVSESTGVALKRSSWLIVLLVLFTVSLSPVQSAPIGQGKTIEAYRAREGYTSICLFVLGSILFIVSCLMK----------
              :* ****. ..    :    *:  *.*: ***::**:*****: :::****.**     *  :   ::*  *** *::*. *. .: *:*
Prim.cons.    2E2ELYL22222P2222222Q222S2G22LKR22WL2VLLVL22222SPVQ2APIGQGKT22222222G22SIC2F22G2IL22222L2KVKRTNSNGIV

                     910       920       930       940       950       960       970       980       990      1000
                       |         |         |         |         |         |         |         |         |         |
Q02004_DUGV   VVVDS--------FGRCPYCNEFTDSLFEEVLHDTLCSLCVCPFCEKQALDLVTLEEHVKECYKVATRKDIFKILGRKFTNALVRREKLFTTGLQLFINK
Q8JSZ3_CCHFV  GLVDSVGNSFFPGLSICKTCSISSINGFEIESHKCYCSLFCCPYCRHCSTDKEIHKLHLSICKKRKKGSNVMLAVCK----LMCFRATMEVSNRALFIR-
               :***           *  *.  : . **   *.  ***  **:*.: : *    : *:. * *  . .:::  : :     :  * .: .:.  ***.
Prim.cons.    22VDS2G2SFFPGLSIC22C2222222FE222H222CSL22CP2C22222D222222H222C2K22222222222222KFTN2222R22222222LFI2K

                    1010      1020      1030      1040      1050      1060      1070      1080      1090      1100
                       |         |         |         |         |         |         |         |         |         |
Q02004_DUGV   TNVVVFALIMCFLLL-LTGHNASAFDSGDLPDGVWEESSQLVKSCTQFCYIEEDVCYCPAEDGVGRKLLFFNGLQNSVKRLSDSHKLLTSVSIDAPWGRI
Q8JSZ3_CCHFV  -SIINTTFVLCILILAVCVVSTSAVEMENLPAGTWEREEDLTNFCHQECQVTETECLCPYEALVLRKPLFLDSTAKGMKNLLNSTSLETSLSIEAPWGAI
               .::  ::::*:*:* :   .:**.:  :** *.**...:*.: * * * : *  * ** *  * ** **::.  :.:*.* :* .* **:**:**** *
Prim.cons.    T222222222C2L2LA222222SA22222LP2G2WE2222L222C2Q2C222E22C2CP2E22V2RK2LF22222222K2L22S22L2TS2SI2APWG2I

                    1110      1120      1130      1140      1150      1160      1170      1180      1190      1200
                       |         |         |         |         |         |         |         |         |         |
Q02004_DUGV   NVESTWKPTLAASNIAMSWSSTDIKGEKVILSGRSTSIIKLKEKTGVMWKLVGSGLASEKKKPFRFPIMDFAQVYNSVFQYITGDRLLSEWPKAVCTGDC
Q8JSZ3_CCHFV  NVQSTYKPTVSTANIALSWSSVEHRGNKILVSGRSESIMKLEERTGISWDL-GVEDASESKL-LTVSVMDLSQMYSPVFEYLSGDRQVGEWPKATCTGDC
              **:**:***::::***:****.: :*:*:::**** **:**:*:**: *.* *   ***.*  : ..:**::*:*..**:*::*** :.*****.*****
Prim.cons.    NV2ST2KPT2222NIA2SWSS2222G2K222SGRS2SI2KL2E2TG22W2LVG222ASE2K2P22222MD22Q2Y22VF2Y22GDR222EWPKA2CTGDC

                    1210      1220      1230      1240      1250      1260      1270      1280      1290      1300
                       |         |         |         |         |         |         |         |         |         |
Q02004_DUGV   PHRCGCQTSTCMAKE--------CHTQECVSTHMVLGIGTGCTCCGMDVERPFNKYLGVKWSTEYLRTEVLVCVEVTEEERHCEIVEAGTRFNIGPITIT
Q8JSZ3_CCHFV  PERCGCTSSTCLHKEWPHSRNWRCNPTWC------WGVGTGCTCCGLDVKDLFTDYMFVKWKVEYIKTEAIVCVELTSQERQCSLIEAGTRFNLGPVTIT
              *.**** :***: **        *:.  *       *:********:**:  *..*: ***..**::**.:****:*.:**:*.::*******:**:***
Prim.cons.    P2RCGC22STC22KEWPHSRNWRC2222CVSTHMV2G2GTGCTCCG2DV222F22Y22VKW22EY22TE22VCVE2T22ER2C222EAGTRFN2GP2TIT

                    1310      1320      1330      1340      1350      1360      1370      1380      1390      1400
                       |         |         |         |         |         |         |         |         |         |
```

```
Q02004_DUGV    ISDPQNIGSKLPESLMTVQEIDDSNFVDIMHVGNVISADNSCRLQSCTHGSAVTTRFTALTALIKDDHSSGLNLAVLDPKVNSSWLSWEGCDMDYYCNVG
Q8JSZ3_CCHFV   LSEPRNIQQKLPPEIITLHPRIEEGFFDLMHVQKVLSASTVCKLQSCTHGVPGDLQVYHIGNLLKGDKVNGHLIHKIEPHFNTSWMSWDGCDLDYYCNMG
               :*:*:** .*** .::*::   :..*.*:*** :*:**.. *:******* .   :.  :  *:*.*: .*  :  ::*:.*:**:**:***:*****:*
Prim.cons.     2S2P2NI22KLP2222T22222222F2D2MHV22V2SA222C2LQSCTHG222222222222L2K2D222G2222222P22N2SW2SW2GCD2DYYCN2G

                     1410      1420      1430      1440      1450      1460      1470      1480      1490      1500
                        |         |         |         |         |         |         |         |         |         |
Q02004_DUGV    DWPTCTYTGVVTQKLREFLKLDQHRKRLHTTLSFSLKKNLSKRSHTSVRLEGKTVTRMEVKVTALIEVDGMELHSKTIRLSGIRLTGLKCSGCFSCTSGI
Q8JSZ3_CCHFV   DWPSCTYTGVTQHNHASFVNLLNIETDYTKNFHFHSKRVTAHGDTPQLDLKARP-TYGAGEITVLVEVADMELHTKKIEISGLKFASLACTGCYACSSGI
               ***:******. ::  .*::* : ..   ..: *  *:  :: . ..: *:.:. *    ::*.*:** .****:*.*.:**::::.* *:**::*:***
Prim.cons.     DWP2CTYTGV2222222F22L222222222222F22K222222222222L2222VT222222T2L2EV22MELH2K2I22SG22222L2C2GC22C2SGI

                     1510      1520      1530      1540      1550      1560      1570      1580      1590      1600
                        |         |         |         |         |         |         |         |         |         |
Q02004_DUGV    SCSVNAKLTSPDEFTLHLRSTSPNVVVAETSIIARKGPSATTSRFKVFSVRDTKKICFEVVEREYCKDCTPDELTTCTGVELEPTKDILLEHRGTIVQHQ
Q8JSZ3_CCHFV   SCKVRIHVDEPDELTVHVKSDDPDVVAASSSLMARKLEFGTDSTFKAFSAMPKTSLCFYIVEREHCKSCSEEDTKKCVNTKLEQPQSILIEHKGTIIGKQ
               **.*. :: .***:*:*::* .*:**.*.:*::***   .* * **.**.  ...:** :****:**.*: :: ..*...:** .:.**:**:***: :*
Prim.cons.     SC2V222222PDE2T2H22S22P2VV2A22S22ARK2222T2S2FK2FS2222222CF22VERE2CK2C2222222C2222LE2222IL2EH2GTI222Q

                     1610      1620      1630      1640      1650      1660      1670      1680      1690      1700
                        |         |         |         |         |         |         |         |         |         |
Q02004_DUGV    NDTCKSKIDCWSNSISSFASGIGDFFKHYIGSIAVGVLGTVLPFALLILFFIYGDKMLWPFKVFCRPCRRCCRKNEG---YNKLAEEEE--LRDIIRKFS
Q8JSZ3_CCHFV   NSTCTAKASCWLESVKSFFYGLKNMLSGIFGNVFMGIFLFLAPFILLILFFMFGWRILF--------CFKCCRRTRGLFKYRHLKDDEETGYRRIIEKLN
               *.**.:* .** :*:.**  *: :::.  :*.: :*::  : ** ******::* ::*:        * :***:..*   *.:* ::**   * **.*:.
Prim.cons.     N2TC22K22CW22S22SF22G222222222G2222G222222PF2LLILFF22G222L2PFKVFCRPC22CCR222GLFKY22L222EETG2R2II2K22

                     1710      1720      1730      1740
                        |         |         |         |
Q02004_DUGV    K---SGELINKDAKDKRTLARLFMSDNPKLKKEKKLSEIA
Q8JSZ3_CCHFV   NKKGKNKLLDGERLADRRIAELFSTKT----------HIG
               :   ..:*:: :   .* :*.** :..          .*.
Prim.cons.     2KKG222L22222222R22A2LF2222PKLKKEKKLS2I2
```

Table S4: Sequence alignments and numbering of different *Peribunyaviridae* M polyproteins. Gn^{cyto} and NSm zinc fingers are highlighted in cyan and magenta, respectively. In grey, additional amino acids used in AlphFold predictions. Red type, green type, and blue type indicate identical amino acid residues, conserved substitution, and semi-conserved substitutions respectively.

```
                               10        20        30        40        50        60        70        80        90       100
                                |         |         |         |         |         |         |         |         |         |
Q806D6_9VIRU_AKAV      MIITILNVLTLVVTVTAMPPRNTNGGRCFYGGDMFRQINSTSPMSEICVRDDISLVKSIGYHKLAANREV-IESSMSYYRLYYVKNWFECNPVQDILGTF
A0A1I9WAL5_9VIRU_CEV   -------MIFMLILFAVAAASPVYQ-RCFQDGAIVKQNPSKEAVTEVCLKDDVSMIKTEARYT--KNSTG-VFSNNVAIRKWLVSDWHDCRPRKVVGGHI
F6JSE3_BUNLC_LACV      -------MIYILVLIAVTAASPVYQ-RCFQDGAIVKQNPSKEAVTEVCLKDDVSMIKTEARYI--RNATG-VFSNNVAIRKWLVSDWHDCRPKKIVGGHI
D7F1G7_9VIRU_BATV      -----------------------------------SRKESQAAISEFCLKDDVSTIKSEIIYE--KNDTG-LFAHNKVLRNWIIKDWKNCNPVPTAGGSI
Q6PN63_9VIRU_NRIV      -----MLLLLVLLIPCYVSASPVVT-RCFHGGQLIAEKKSQTAVSEFCLKDDVSTIKSEITYE--KNNTG-LFAHSKVLRNWIIKDWKNCNPVPTAGGSI
GP_BUNYW_BUNV          ----MRILILLLAVTQLAVSSPVIT-RCFHGGQLIAERKSQTSISEFCIKDDVSMLKSEIVYT--KNDTG-IFGHSKVFRHWTITDWKACNPVVTAGGSI
A0A0R7FK51_9VIRU_UMB   ------MMVFALLLISLAGLSSQATIRCFSGGSKIMERTSNVSLSELCLKDDVSQLKIIVDHT--KNDSG-LFSTASVWRKWIVSDWTTCNPKKMALGTI
A0A0R7FK55_9VIRU_WIT   ----MGFLNWLLLFFAMHTVRAIPSTRCFAGGQKMHELTSSKVTSEACLKDDVSQVKIHTNYF--KNETG-IYAIMTAYRKWTVADWHECRPKKTVSGSI
A0A2I4SI68_9VIRU_TCM   -----MELILFVTMIAAVLANPTPE-RCFTGGTKLLESKSPQAMTYVCLKDDISYVKSEVVSTDEKDKNGRTLFLNTVFRKIQIDDWQNCRPEKQVGGPI
Consensus                                        RCF gG    #  S    se ClkDD!S vKse      k#  g   s     Rkw ! #W  CnP k  gG i
Prim.cons.             MIITMM6LILLLLLIA3AAASPV2T3RCF2GGQKI2E3KSQ2AVSE2CLKDDVSMIKSEI2YT22KNDTGR2FSHNKV4RKWIV2DW2NCNPVKT2GGSI

                              110       120       130       140       150       160       170       180       190       200
                                |         |         |         |         |         |         |         |         |         |
Q806D6_9VIRU_AKAV      MVFDVNHEGILAPKTYACRATCSISLERDTGNVVLESPALNHYTIHGTTIKNGWFKTKVSIDLDNTCEDLHITCGHKTLNVHACFRQHKSCIRYFKGSIL
A0A1I9WAL5_9VIRU_CEV   NVIEVGDDLTLHTESYVCSADCTIGVDKETAQVRLQTDTTNHFEIAGTTVKSGWFKSTTYISLDQTCEHLKVSCGPKSIQFHACFNQHMSCVRFLHRTIL
F6JSE3_BUNLC_LACV      NVIEVGDDLSLHTESYVCSADCTIGVDKETAQVRLQTDTTNHFEIAGTTVKSGWFKSTTYITLDQTCEHLKVSCGPKSIQFHACFNQHMSCVRFLHRTIL
D7F1G7_9VIRU_BATV      NVIEVNTDLSLTTKTYVCSRDCTITVDKEDAQIIFQTEKLNHFEVSGTTLSSGWFKTKASVTLDRTCEHIKVTCGKKILQFHACFKHHMSCIRFFHGTIL
Q6PN63_9VIRU_NRIV      NVIEVNTDLSLTTKTYVCSRDCTITVDKEDAQIIFQTEKLNHFEVSGTTLSSGWFKTKASVTLDRTCEHIKVTCGKKTLQFHACFKHHMSCIRFFHGTIL
GP_BUNYW_BUNV          NVIEVDKNLNLVTRNYVCTGDCTITVDRKNAQIIFQTDKLNHFEVTGTTISTGWFKSKASVTLDRTCEHIKVSCGKKTLQFHACFKQHMSCVRFLHRSIL
A0A0R7FK51_9VIRU_UMB   SVIDIDKDFILKSSSYICNKECTITVDKERAQVLLQSDGLNHFEVSGTTLSSGWFKLTATVTLDQTCEHVKVSCGQKSMQFHACFKNHMSCIRFLHRSIM
A0A0R7FK55_9VIRU_WIT   NVIEVDKSMMISSVVYTCDKDCTINIDKENAQIILHTEGINNFEVSGTTVIKAWFKTTATIPLQQTCEHIKIICGKKILQFHSCFKHHMSCIRYLQNSMV
A0A2I4SI68_9VIRU_TCM   MIISFDEHGSMESEDYVCKNDCEIKLDRDAGLINLETSTLNYYQISGTTKVSGWFKTRTSISLKHTCENIKIQCGEKSILLHACFKNHMECYQFLISHML
Consensus               !i vd    l    YvC  dCtI  #r# aq! l t  lNh%e!sGTT  sgWFKt  s! Ld TCEh k! CG K lqfHaCFk HmsCir%l  sil
Prim.cons.             NVIEVDKDLSL2T22YVCSADCTITVDKETAQIILQTD2LNHFEVSGTT2SSGWFKT2ASITLDQTCEHIKVSCGKKSLQFHACFKQHMSCIRFLHR2IL

                              210       220       230       240       250       260       270       280       290       300
                                |         |         |         |         |         |         |         |         |         |
Q806D6_9VIRU_AKAV      PEVMIESICTNAELILLCCFSAISCFVAIILTKTYLVYLLIPIFYPFVKLYGLLLQRFCKQCKNCLLPIHPFSPCPTTCICGMVYNSTEALKVHRKCLNC
A0A1I9WAL5_9VIRU_CEV   PGSIANSICQNVEIIILVTLALLIFILLSIISKTYICYLLMPIFIPIAYIYGLIYNKSCKKCKLCGLVYHPFTECGTHCVCGARYDTSDRMKLHRASGLC
F6JSE3_BUNLC_LACV      PGSIANSICQNIEIIILVTLALLIFILLSILSKTYICYLLMPVFIPIAYVYGVIYNKSCKKCKLCGLVYHPFTECGTHCVCGARYDTSDRMKLHRASGLC
D7F1G7_9VIRU_BATV      PGTMATSICQNIELIIIISLTLIIFILMVILTKTYICYLLMPLFMPIAYFYGWSYNKSCKKCPCCGLAYHPFTNCGSHCVCGLKFEASDRMRIHRESGLC
Q6PN63_9VIRU_NRIV      PGTMATSICQNIELIIIISLTLIIFILMIILTKTYICYLLMPLFMPIAYFYGWSYNKSCKKCSCCGLAYHPFTNCGSHCVCGLKFEASDRMRIHRESGLC
GP_BUNYW_BUNV          PGSMAISICQNIELIIITILALCIFIIMIILTKTYICYVLIPVFMPIAFAYGWAYNRSCKKCTCCGLAYHPFTNCGSYCVCGSKFETSDRMRMHRESGLC
A0A0R7FK51_9VIRU_UMB   PASFAVSICQNVELILMTTFTLIIFLLLSILTKTYICYILLPIFIPFAYAYGFIYDKTCKKCSLCGLAYHPFTKCGIHCVCGCRFENSDAMKTHREKGKC
A0A0R7FK55_9VIRU_WIT   PGKISNAICQNLELLIMTTITTLIFSILILLAKTYLCYLLLPLFIPIAYCYGFFYDRSCKKCKSCGLAYHPFTKCGEHCVCGGKFETSERMKIHRDSGLC
A0A2I4SI68_9VIRU_TCM   PKTFSSSVCANLEIIILTAFSIIAFLLLSILTKTYICYILLPFFIPCTYIYSKLYMKCCKICNNCGLRYHPFSPCGKICVCGSVYDSSERMKIHRQTGLC
Consensus              P     s!CqN Eliilt f  iif ll iLtKTYicYlL P FiP ay Yg  y k CKkCk CgL yHPFt Cg  C!CG  %# s#r$k HR  glC
Prim.cons.             PGSMANSICQNIELIIILTTLTLIIFILL2ILTKTYICYLLMP2FIPIAY3YGWIYNKSCKKCK2CGLAYHPFTNCG2HCVCG3KFETSDRMKIHRESGLC

                                                      BUNV NSm 1        10        20        30        40        50
                                                               |         |         |         |         |         |
                              310       320       330       340       350       360       370       380       390       400
                                |         |         |         |         |         |         |         |         |         |
Q806D6_9VIRU_AKAV      TGYKTLTKTRYLCKKRLPNMVLATISTVLFFTFITPITA--------ECYNYTSLPADFQAAIDKNN----SCTREQLIMIILTILCIASTIMVISAVHL
A0A1I9WAL5_9VIRU_CEV   PGYKSLRAARVMCKSKGPASILSIITAVLILTFVTPINAMMVG-ESKETFELEELPDDMLEMANNLNTYYMLCVANYAVTWSLLIVALIIGLLFKKYQHR
F6JSE3_BUNLC_LACV      PGYKSLRAARVMCKSKGPASILSIITAVLVLTFVTPINSMVLG-ESKEVFELEELPDDMLEMALRINFYYFTCILNYAVSWSLVIIGLLTGLLFKRYQHK
D7F1G7_9VIRU_BATV      QGYKSLRVARLLCKSKGSSLIISILLSMLILSFVTPIEGTLTNYPESRKYDLEEIADVLEGFVVEKGIKEYVVFYTSIFGALFLLMALIVIIALNRVTEY
Q6PN63_9VIRU_NRIV      QGYKSLRVARLLCKSKGSSLVISILLSMLILSFVTPIEGTLTNYPESRKYDLEEIADVLEGFIVEKGIKEYVVFYTSIFGALLLLIALVITITLNKVTEY
GP_BUNYW_BUNV          QGFKSLRVARRLCKSKGSSLIISILLSVLILSFVTPIEGTLTNYPTDQKYTLDEIADVLQAKTHEDSTKYYIILYTSLFGAGLTIIFAGVALGLTIILEV
A0A0R7FK51_9VIRU_UMB   PGFKSMRTARVLCKAKGSAFTLSVLLATLALGFLTPVQGLILD-ETQASCTEDQLPESFQVMQSTISVFKFATIMNAGWTYCMFLITLALYVIYKKYAHK
A0A0R7FK55_9VIRU_WIT   QGYKSLRSARVLCKSKASALTLSVLLSILLLGFLTPVQGVPI----KTNFTIDELPEYSQDQSNQVTLLHKNVIYSLLVDLLLIILVFVSIICINHFVYS
A0A2I4SI68_9VIRU_TCM   PGYKYMMSARFMCKSKGWNMIVAILLSVLFFSFITPIGA------NSTCYTLDEFPDMYINSIKNMEFIKAYLFYWLAVNSLLLLIFITVIITIHYFEHI
Consensus               G%Ks$r aR $CKskg    lsill vL l F TP! a          ytldelpd  q  i                   $ ii     i      h
Prim.cons.             2GYKSLRVARVLCKSKGSALILSILLSVLILSFVTPIEGTLTNY232EKYTL2ELPD2LQ3MI2E2N3KYYV22Y2S2VGALLLIIAL3VII2L2KY3H2

                       60        70        80        90       100       110       120       130       140endNSm
                       |         |         |         |         |         |         |         |         |  |
                              410       420       430       440       450       460       470       480       490       500
                                |         |         |         |         |         |         |         |         |         |
Q806D6_9VIRU_AKAV      YIRIYYVFCAYCGMVHEKKGLILLDNFTSHCLTCICRDKS------IHRANNNCVTPMKYKTAKYTNLLCIILLIIIAVTPAFSA-------CIQEKEIE
A0A1I9WAL5_9VIRU_CEV   FLDIYAMYCEECDMYHDRSGIKWNGDFTNKCGHCTCGQYEDASGLLVHRKTYNCLIQYKSKWIMNFLVTYVILVLLKDSVVIVQAKGVDFSECIEAEKVR
F6JSE3_BUNLC_LACV      FLNIYAMYCEECDMYHDKSGLKRHGDFTNKCRQCTCGQYEDATGLMTHRKTYNCLVQYKAKWIMNFLIIYIFLVLIKDSAIIVQAAGTDFSSCLETESIN
D7F1G7_9VIRU_BATV      LTNINVLYCHECSMYHSKKNIKYIGDFTNKCGFCTCGELEDQEGLKIHKVSRKCIYKYQLTWSKVIMMILVCLLIAQNTILIVAAS----DDCWTKKSLE
Q6PN63_9VIRU_NRIV      LTNINVLYCYECSMYHSKKNIKYIGDFTNKCGFCTCGELEDQEGLKIHKVSRKCIYKYQLTWSKIIMTILVCLLIAQNTILIVAAS----DDCWTKKSLE
GP_BUNYW_BUNV          LTKINVIFCNECNMYHSKKSIKYVGDFTNKCGFCTCGLLEDPEGVVVHKAKKSCTYSYQINWVRGIMIFVAFLFVIQNTIIMVAAE----EDCWKNEELK
A0A0R7FK51_9VIRU_UMB   LLNYYVIHCKECDMLHDKSGLKYNGDFTNKCGFCTCGQVEDAMGLTVHQIRNHCSANYRTRMVLNWMAVLLLATIIKDSIMLAAAAGTEFETCVKKVEMT
A0A0R7FK55_9VIRU_WIT   IANIFAIYCNTCDMYHSRSNLKYFGDFTNRCGSCTCGQFEDMQGVLIHKRSSRCLTKYKIMTLKHVILWIIITLVVKDFAMVAQAK--TLKECLQIKELE
A0A2I4SI68_9VIRU_TCM   YLRPYIFKCEDCKMFHSKHRLVIEEFGTSRCGSCTCGCPDDPPMVHFHQTSRQCVLKYIVKILKTYSICIILYCFISSTTIGAIAT--EVGVC-DDDPID
Consensus                 iy   C  C M H k glk  gdfTn Cg CtCg  ed  g   H     C   yk k  k      i l  i        A       C    ei
Prim.cons.             LLNIYVIYCEECDMYHSK2GLKY2GDFTNKCGFCTCGQLEDAEGL2IHK3SRNCLYKYK2KW4KNIMIILIILLIIKDTIIIVAA3GT2F3DCW4KKELE

                              510       520       530       540       550       560       570       580       590       600
                                |         |         |         |         |         |         |         |         |         |
Q806D6_9VIRU_AKAV      T-IDDAAMCIGLYQNITQPKEYNTFVKELSSTLSSHEIEF-LLPAVKPSFDYLITKSSTSKDLHSATIHEFIAANLYPNNFKKHLASAGPGSIQWRTYIQ
A0A1I9WAL5_9VIRU_CEV   WNCTGPFVNLGNCEKKQKKEPYSNIAAQLKGLGAISILDVPIIASIPEDIAGALRYIEDQRSYHSQLTVEYAMLTRYCDYYTQFSDNSGYSQTTWRVYLR
F6JSE3_BUNLC_LACV      WNCTGPFLNLGNCQKQQKKEPYANIAAQLKGLKAISVLDIPMITEIPDDIASALRYIEEREAFHVQLTAEYAMLTRYCDYYTQFSDNSGYSQTTWRVYLR
D7F1G7_9VIRU_BATV      MECIGPLQQVDTCDDKTSRS-YSGEAKKLVRASKISDLDAAQVGLLGSTIESAIASIRRQRTYSTMHLLESVFLGKHCDYYKVFEHNSGYSQAKWRLTAK
Q6PN63_9VIRU_NRIV      MECIGPLQQVDTCDDKASRS-YSGEARKLVRASKISDLDAAQVGLLGSTIESAIASIRKQRTYPTMHLLESVFLGKHCDYYKTFEHNSGYSQAKWRLTAK
GP_BUNYW_BUNV          EDCVGPLIAPKDCTDKDHKT-YLSEASLLATAKKITQVDAENVEILGKTMESAIRVIERQKTYHRMHLLEAVFLNKHCDYYKMFEHNSGYSQVKWRMMIK
A0A0R7FK51_9VIRU_UMB   PECTGPFMEVGICDHQQKSLKYHVVANMLLSKGTISKLDVDIIDALPDKIAGDLQMIQSSSDFHHQQMLEYVFLLRHCDYYSSFVSDSSYSQILWQTAAK
A0A0R7FK55_9VIRU_WIT   KDCTGPFFDIKECSTTTMQKSYGDIANWLKTKEVISELDKKAASELSQDVTRSLQKIETTLGLHQKILFEILFLSRNCDYYKSFDNVYSTHQLQWQTLAK
A0A2I4SI68_9VIRU_TCM   TECWGLNIEKDIISNRNDIN-----GTLRKMFPDMTEEELNIAHTDYATYPDFLIEARSYDALRPKQILEAVYFSRNSFHHITGADQRYY--ISWKLRTR
Consensus                c gp    g c        y   a  l     i e #              l  i     lh     E   l r cdyyk f    gy qi Wr
Prim.cons.             3ECTGPF33VG3CDDK2KK3PYS22A33LK4A3KISELDA2I2G3L22TI2SALR3IE3QRTYH22HLLEYVFL4RHCDYYK3FE2NSGYSQIKWR2YAK

a.bockmann@ibcp.fr            610       620       630       640       650       660       670       680       690       700
                                |         |         |         |         |         |         |         |         |         |
Q806D6_9VIRU_AKAV      NNNLHLCNDHVVKMICRCVIKQEECSS-TKVDDGEQIAQYYKRNKEFYKADLEILYTVISRAIPGLVGNLLRQVLKSQKYGESLHVLNKIKKDVSKN---
A0A1I9WAL5_9VIRU_CEV   SHDFEACVLYPNQHFCRCVKHGDKCSS-SNWDFASEMKNYYSGKQSKFDKDLNLALTSLHHAFRGTSSSYIAGLLSSKANDELVQYTSKIREKFPGNALL
```

```
F6JSE3_BUNLC_LACV     SHDFEACIMYPNQHFCRCVKNGEKCSS-SNWDFANGMKDYYSGKQAKFDKDLNLALTALHHAFRGTSSAYIATMLSKKSNDDLIAYTNKIKTKFPGNALL
D7F1G7_9VIRU_BATV     THHFDICSRHSTHHFCRCISDGTKCQN-GDWDFAGEMNSTYQSKKDFFEHDFKLFCTLVENAFPGTTESLFYEMLSKKNTTGITKLLDKLTKKFGNNNMF
Q6PN63_9VIRU_NRIV     THHFDICSRHSTHHFCRCISDGTKCQN-GDLDFAGEMNSTYQSKKDFFEHDFKLFCTLVENAFPGTTESLFYEMLSKKNTTGIKQLLDKLTRKFGNNNMF
GP_BUNYW_BUNV         TQHFDICALQANSPFCAQCIADNSCAQ-GSWEFDTHMNSTYSSKVDNFKHDFSLFLRIFEAAFPGTAYVHLLTNIKEKKPYQAVSMIEKIKKKFPNNKLL
A0A0R7FK51_9VIRU_UMB  LNETNLCKHESSKKSCKCLKSNANCGE-SE-DINTAAQTYYKNKESELGQDLTTIMYILKKMIPGSGYSYLLNATKHNKLELFKAYMTNISSKYPNNKKL
A0A0R7FK55_9VIRU_WIT  NGDFDYCKTKGTEALCKCI-SGAACQS-II-DKKDDIDTHYKSKDQQRKKDIETVMSITRYMFPGTGHSYIANQTVTKQYTKVVEYFTSFAEKHATNLRL
A0A2I4SI68_9VIRU_TCM  VLELNTCKLVAKPYPCKCISIEEECTEFALKKFRSANLDNYLTDQTKLNWDRLKLQDAMYEFMPPTVVHKYISIVESKNKTMIDGFSHDIYQMYTN----
Consensus                   C        C C    e C s    df      yY  k      Dl         a pgt         sk           ki  k   n
Prim.cons.            TH2FDICKLHS23HFCRCI22GEKC2SF22WDFA3EMNSYY2SK2D2FK2DL3LFLTILE2AFPGT43SY2A3MLS2K23T2IV2YL3KIKKKFPNN3LL

                             710       720       730       740       750       760       770       780       790       800
                               |         |         |         |         |         |         |         |         |         |
Q806D6_9VIRU_AKAV     -------NQLNGIVEFLIHINSQNITEEVRELRIRPDLSIRGSKFTDKN----PGIPDIKECQTPLFITCTGKRFR-SLMKQYIAC-SNGGVKLYQRPNK
A0A1I9WAL5_9VIRU_CEV  KAVIDYIAYMKGLTEMSNFKKDEFWDELVYVPDPTKPPKLSRSSGTVYDFKSATSNLGEKNCKDVKGVVCLSPRSG-VTYDSIIACGEGSSPNIYRIPKT
F6JSE3_BUNLC_LACV     KAIIDYIAYMKGLPGMANFKHDEFWDELLYKPTPTKAPNLARGRESSYNFKLAISSKSIKTCKNVKDVACLSPRSG-AIYASIIACGEPNGPSVYRKPLN
D7F1G7_9VIRU_BATV     VGIWKFGQYLMSLPYVNETSLSPIQLAKVLEVTDQHHRSVSGRQESLAS---ATPGSKSSECDHAKKVSCISPRFG-APMEGLMACGNSPNYKIYKTPAK
Q6PN63_9VIRU_NRIV     VGIWKFGQYLMSLPYINETSLTPAQISKILEVTDQHHRSISGRQESLAS---ATPGSKSKECGHAKKVSCISPRFS-VPMEGLMACGDSPNYKIYKTPTK
GP_BUNYW_BUNV         IGYLDFGKYLLGLSHASTYELQQRQLDKLYQPTELTRSG--GQQTSLAN---SVVGQATKECKKYKDVSCLSPRFG-IPLEDLISCCDQPNYNIYKKPKK
A0A0R7FK51_9VIRU_UMB  KDFMAITNAILANLSALSHSSEESIPEYKQIDFSSSVGETTDMLEANFN---QTISTDKITCENPKVMICISPRTK-ISSGELYVCQRSGKYYIIDTGRF
A0A0R7FK55_9VIRU_WIT  KAFSILISGLLKTTTSLE--PPEQLASFVPKLARPTIPGFTDLIDSNFD---QSAQNDGRRCKSPSVIVCASPRTG-YTSDEMYIC-RDTKYYIIDTKGL
A0A2I4SI68_9VIRU_TCM  --YPYILNFMYLLMNITSRSQDFSISALLQNQTHATTQILNRGLVTIES----LELGYRKTCKNPKRYFCKSERVGLVATKPLLVCDRDTSNGETKRTLH
Consensus                    n l  l           i e                  t  n          k Ck pk   C spR g         C        y  p
Prim.cons.            K2I2DF2NYLLGLP23LE3SLDE222E2VYEPT22THPSLSG3QESLANFK2AT2GSD2KECKNPK3VSC2SPRFGLVPME3LIACGRSPNYKIYKTP2K

                             810       820       830       840       850       860       870       880       890       900
                               |         |         |         |         |         |         |         |         |         |
Q806D6_9VIRU_AKAV     PLA-----LVGNKLCIGDKYCMIAFDPMVIDE--NIQKLDCYSLAATDQSDGMLKPE-RSIRLLKTGECKIAGA--LSRIAVSINQKNYKYSTIVHKKSG
A0A1I9WAL5_9VIRU_CEV  GVYQ--SNSEPSNYCVSDSHCLEDFEVINQEELDAIKKSRCWEVDYLYANPSKQSDGIRSCRMRDAGHCNVTTNR-WPIIQCDDNKYYYSELQRDYDKEQ
F6JSE3_BUNLC_LACV     GVFQ--SSTDQSMYCLLDSHCLEEFEAISQEELDAVKKSKCWEIEYSDIKPLQESDGTKSCRMKDSGNCNVATNR-WPVIQCENDKFYYSELQKDYDKTQ
D7F1G7_9VIRU_BATV     -LYK--SNNKGEVWCSGDVHCFQELTPASQESVDRIKQMTCFLTE-PEVNDDVFSIAIAMCKVQDKGVCTINEDR-WNIIKCDSGLIYYTDQREGQDTGN
Q6PN63_9VIRU_NRIV     -LYK--SNNKGEVWCSGDVHCSQELSPASQESVDRIKQITCFLTE-PEVSDDVFSIAISTCKVQDKGVCTVNEDR-WNVIKCDSGLIYYTDQRDGQDTGN
GP_BUNYW_BUNV         -VYK--AHDKEETWCINDQHCLVDFVPAEADTVEKLKPMKCWLVD-PGKNDDVYSIAIKTCRVVDKGVCTVNSQK-WNIIKCDSGPLYYSDHIPGEDTGN
A0A0R7FK51_9VIRU_UMB  KLYK--LIGAGNLYCAADAQCRYEFRILTTGEMQATFKDN-CKASVHTGTPGYQNDAVTHCKVLASGKCKYNNQTLIDITLCANNRYYPEAKGRI--FGV
A0A0R7FK55_9VIRU_WIT  QLYK--LDNSAGTYCVGDKYCNLKYRVLTPEESLALRKDNNCKHKEYTEPKNYLTERLTICRQKATGKC--GEEPTFRVSLCENGFVYPTAAKASPKDSA
A0A2I4SI68_9VIRU_TCM  RWSQDLVEVDQSIICRKDYTCSSYFDNSTDIELQTITKKKQRCNEIAKVYDPVYNQAISKCKMVEKGFCNFNGNP-KQIVKCSDNTLVEQIATGNYLNDM
Consensus              l            C  D  C   %      e   i k  c         d         cr    G C         ii c  n  yy
Prim.cons.            GLYKDLSNNKG22YC3GD3HCL2EF3PA2QEE2DAIKK3KCWL2EYP3VNDDV4S2AI4SCRV4DKGVC2VNENR2WNIIKCD2223YY2D33RGYD2GN

                             910       920       930       940       950       960       970       980       990      1000
                               |         |         |         |         |         |         |         |         |         |
Q806D6_9VIRU_AKAV     LVDEYCLSPNCDLDCYPYYPANLVDCSWSESTHSTLNQKVISHTDIESFISSVKLSLHNDLIQHHFRPLSNMPHVKPNFKSINVQGTISGGKIQDSYITF
A0A1I9WAL5_9VIRU_CEV  DIGHFCLSPRCTTVRFPIHPKHVSNCDWQVSHSTVDKVDVHNLEDIEQYRKAITQKLQNSLSIFKYARTKNLPHIKPIYKYITIEGTETAEGIESAYIES
F6JSE3_BUNLC_LACV     DIGHYCLSPGCTTVRYPINPKHISNCNWQVSRSSIAKIDVHNIEDIEQYKKAITQKLQTSLSLFKYAKTKNLPHIKPIYKYITIEGTETAEGIESAYIES
D7F1G7_9VIRU_BATV     DFGEYCLSHSCRIERFPINPAIISDCLWEYHSRKSKYITSLDLESLEEFKRAISEKLSHTLVVYNFKPTANLPHIKPVYKYITVQGVENSDGVDSAYITA
Q6PN63_9VIRU_NRIV     DFGEYCLSHSCRIERFPINPAIIRDCLWEYHSRKSKYITSLDLENLEEFKRAISEKLSHTLIVYNFKPTANLPHIKPVYKYITVQGVENSDGVDSAYIAA
GP_BUNYW_BUNV         DIGHYCVSAGCKTDRYPINPDVVTDCVWEFTSRKSQYIGKISMQSLEDYEKALTDRLTHTLETYSFAPLENLPHIKPVYKYITAQGVENSDGIEGAFITA
A0A0R7FK51_9VIRU_UMB  DPEDLCFDSACTKQQTPYNEELISACVWENPTLRPIRPRTEVSTTFQEYKDQLLKKINTDLVLHKFVRTQNLPYFIPQFKYITIQGTATTDGITSSFIFF
A0A0R7FK55_9VIRU_WIT  FPNDRCFDTTCKYGYYPYNMIQYSKCVWDNIKITSSRIKIASYENFQEYKDQLLKKITSDLTIGKFNLVENMPYFIPKRKYLTIKGVTTADGIDGSFVEF
A0A2I4SI68_9VIRU_TCM  KMDQVCFKTDCGDVK-RIHPDSLTNCTINVPRQLPVHIDRVDTNDFKIYKAHLEEDFLTTLSKFYFMPTKGLPHIVPDFRPIYLRGTETTDGLESSYFEI
Consensus                  C s  C    ypi p     C w         i      d e %k  l   l   L    % pt n$PhikP fkyit qGtet dgi ss%i
Prim.cons.            DIG2YCLSP2CTTVRYPINPA2ISDCVWEVSSRKSKYIDV32LED3EEYKKA2TEKL32TLS32KFAPTKNLPHIKPVYKYITIQGTET2DGIESAYIE2

                            1010      1020      1030      1040      1050      1060      1070      1080      1090      1100
                               |         |         |         |         |         |         |         |         |         |
Q806D6_9VIRU_AKAV     SIPLMTGLSQGFTLQDHKGNTLFDIIAYVKSARVIATYNHEYKTGPTVSINVQHNEQCTGSCPSSIPKKDN-WLTFSREHTSTWGCEEWGCLAIGTGCVY
A0A1I9WAL5_9VIRU_CEV  EIPALAGTSIGFKINTKEGKHLLDVIAYVKQASYSSLYNKMYVTGPTVGINTKHDELCTGPCPVNVPHSDG-WLTFARERTSSWGCEEFGCLAISDGCVF
F6JSE3_BUNLC_LACV     EVPALAGTSIGFKINSKEGKHLLDVIAYVKSASYSSVYTKLYSTGPTSGINTKHDELCTGPCPANINHQVG-WLTFAKERTSSWGCEEFGCLAVSDGCVF
D7F1G7_9VIRU_BATV     SMPALSGTSIGYNIMSRDNFPLFDIIIFIKSAIIKATYNHIYDTGPTIGINVMHDEHCTGQCPTDIPHKEN-WITFAQERTSRWGCEEFGCLAVNTGCVF
Q6PN63_9VIRU_NRIV     SMPALSGTSIGYNIMSRDNFPLFDIIIFIKSAIIKATYNHIYDTGPTIGINVMHDEHCTGQCPTDIPHKEN-WITFAQERTSRWGCEEFGCLAVNTGCVF
GP_BUNYW_BUNV         SIPAAGGTSIGYNVRSKDGFPLLDLIVFVKSAVIKSTYNHIYDTGPTISINTKHDEHCTGQCPSNIEHEAN-WLTFSQERTSRWGCEEFGCLAVNTGCVF
A0A0R7FK51_9VIRU_UMB  EIPALTGASVGYKVVNNEGQELIDMVVYIKTSKTSAKYKRVYYTGPTIAINTKHQEHCTGTCPERIEHDDG-WATFSKERTSNWGCEEFGCLAVETGCLF
A0A0R7FK55_9VIRU_WIT  EIPSLTGVSAGYTVTTKEGQELFDFIVYIKNSATSATYSYAYSTGPTIGINNKHTEVCTGKCPEKIPHEAG-WATFSRERTSTWGCEEFGCLAINEGCLY
A0A2I4SI68_9VIRU_TCM  EMIALSGKATGIKLYASNDLYLFDVVIYVQAANVTSTYVPIYRTGPTLTFNVKHEEICTGLCPSDLPRADNTWMTFSKESTSTWGCEEFGCLAIGEGCVY
Consensus             eipa$ G s G       g  LfD ! %!k a   atY   Y TGPT  iNvkH E CTG CPs iph dn W TFs ErTStWGCEEfGCLA! tGCv%
Prim.cons.            EIPAL2GTSIGYKI2SKEGFPLFD2I3YVKSA2ISATYNHIYDTGPTIGIN2KHDEHCTGQCPS2IPHKDNTWLTFS3ERTS2WGCEEFGCLAVNTGCVF

                            1110      1120      1130      1140      1150      1160      1170      1180      1190      1200
                               |         |         |         |         |         |         |         |         |         |
Q806D6_9VIRU_AKAV     GSCQDVIREEATVISRVNNEQLEVEFCVSEPTSTMCNTINVLEPVLGEHMQFEVHSVQTNLLPEVALIKNRRVYKGSINKKGVFNPQCGSVQSFDGKLYG
A0A1I9WAL5_9VIRU_CEV  GSCQDLIKEELAVYRKETEEATNVELCLTFSDKTYCTELNPITPIITNLFEVQFKTVETYSLPRVVAVQNHEIRIGQINDLGIYSKGCGNVQKVNNTIYG
F6JSE3_BUNLC_LACV     GSCQDIIKEELSVYRKETEEVTDVELCLTFSDKTYCTNLNPVTPIITDLFEVQFKTVETYSLPRIVAVQNHEIKIGQINDLGVYSKGCGNVQKVNGTVYG
D7F1G7_9VIRU_BATV     GSCQDIIRPETKVYRKAVEESVLLTVCITYPGKTFCTEINAIEPKITDELELQFKTVDTKTLPNILAVQNHKLYSGQINDLGSFSQGCGNIQKTNSSIIG
Q6PN63_9VIRU_NRIV     GSCQDIIRPETKVYRKAVEESVLLTVCITYPGKTFCTEINAIEPKITDELELQFKTVDTKTLPNILAVQNHKLYSGQINDLGSFSQGCGNIQRTNSSIIG
GP_BUNYW_BUNV         GSCQDVIRPETKVYRKAVDEVVILTVCITYPGHTFCTEINAIEPKITEEIELQFKTVDTKTLPYIVAVNNHKLYSGQINDLGTFGQMCGNVQKTNSSILG
A0A0R7FK51_9VIRU_UMB  GSCQDVIRRELDVFSKLEEDKILTELCITLSHDTYCVTIDSTTPTINDYFEIQYKTVDVQTLPKMIGVTHHKLLRGQINELGSFGKFCGNIQVVGNQTYG
A0A0R7FK55_9VIRU_WIT  GSCQDVVKPELDIYSKAGADTTKTNVCISLNHKTYCQEIDALNPIITENIEAQFKTVESKNLPRLIAIKNHKLYTGQINELGSFGKYCGNLQVTNNQTLG
A0A2I4SI68_9VIRU_TCM  GQCKNVIKPESEVWEKQSEERITLNLCITTSYEYYCKEIESTVPLITNKIEAQFNTIESFKHPERILIRNHIAFQGQINGLREYNSYCGNVQIVGSTTMG
Consensus             GsCq#!!r E  !  k  e#    e Cit    tyC ei#   P it#  # #fkt!#t  lP    ! nh  y GqIN lg %   CGnvQ      yG
Prim.cons.            GSCQDVIRPELKVYRKAVEE22LLE2CITY2GKTYCTEINAIEP2ITDE2ELQFKTV2TKTLPRI2AVQNHKLYSGQINDLGSFSKGCGNVQK2NS2IYG

                            1210      1220      1230      1240      1250      1260      1270      1280      1290      1300
                               |         |         |         |         |         |         |         |         |         |
Q806D6_9VIRU_AKAV     IGNPKFDYICHALSRKDIVVRKCYENHYYSCATLKE-AVEIKPNITNSKTMLYNDNALLGSASVKIMLGDLIYQQTSVQEKDIRGHATCGGCTDCFNDVA
A0A1I9WAL5_9VIRU_CEV  NGVPKFDYLCHLASRKEVIVRKCFDNDFQACKFLQS-PPSYRLEEDDGSITVIDYKKILGTIKMKAILGDVKYKTFA-ENIDITAEGVCTGCVNCFENIH
F6JSE3_BUNLC_LACV     NGVPRFDYLCHLASRKEVIVRKCFDNDYQACKFLQS-PASYRLEEDSGTVTVIDYKKILGTIKMKAILGDVKYKTFA-DNVDITAEGSCTGCINCFENIH
D7F1G7_9VIRU_BATV     TGTAKFDYVCHGASRKDIIVRRCYNNNYESCKLLKE-EQSLMFADNHETIDVANVRHLLGDLQFKLMLGDLRYKSFA-ENPDLEIEAKCVGCPSCFTSYS
Q6PN63_9VIRU_NRIV     TGTAKFDYVCHGASRKDIIVRRCYNNNYESCKLLKE-EQSLMFADNHETIDVANVRHLLGDLQFKLMLGDLRYKSFA-ENPDLEIEAKCVGCPSCFTSYS
GP_BUNYW_BUNV         TGTPKFDYTCHGASRKDIIVRRCYNNNFDSCKLLKE-ETQLIFNDDHDTITVYNTNHLIGELAIKLILGDIQYKLFT-ETLDLQIDAKCVGCPDCFESYS
A0A0R7FK51_9VIRU_UMB  QAAVKFDYICHAAQRKDVVIRKCLENNFQSCKLLTH-DTDLILEESETSVEVINNKRITGQIALKVLLGDFNYKQYT-ANVEADIEANCVGCTNCIKGIT
A0A0R7FK55_9VIRU_WIT  LADVKFDYTCHAAQRKDILIRKCLENSYQSCKLLKE-ESDYMLIEEATHVTISDNKKINGNLAIKAIFGDFNYKSYT-KDLDFEAEISCVGCFSCLHGIV
A0A2I4SI68_9VIRU_TCM  MGIPKMDYKCHAASRKDVIVRHCYDNNYLSCLELKPLPKDMIEYETDKLLTLNTHNKITGTLKLKIKLGDIQYKLFQ-EDTKLDITGECVGCIDCIDGIT
Consensus              g pKfDY CHaasRK#!i!RkCy#N % sCk Lke        e     t  n n i G    K  lGD  Yk f   # d    a CvGC dCf  i
Prim.cons.            TGTPKFDY4CHAASRKDIIVRKCY3NNYQSCKLLKELE2SL2LEEDH3TITVIN3KKILGTL24K2ILGDL4YKSFAVEN3DLEIEAKCVGCP3CFE2IS

                            1310      1320      1330      1340      1350      1360      1370      1380      1390      1400
                               |         |         |         |         |         |         |         |         |         |
Q806D6_9VIRU_AKAV     CKISMTSNGVYQCPIVSSCDSYINNVYINEGTNDVNLKFRCLKA----EIKISICGKEIPVKSEIIKDTKKLDWASADQTSYIKEFDKKCATWLCRAYNE
A0A1I9WAL5_9VIRU_CEV  CELTIHTTIEASCPVTSPCTLFHDRILITPDEHKYAMKVICTEK-QTGTLPFKICNSKVDATLTLVEAKPILELASVDQTAYIREKDERCKTWMCRVRDE
F6JSE3_BUNLC_LACV     CELTLHTTIEASCPIKSSCTVFHDRILVTPNEHKYALKVVCTEK-PGNTLTIKVCNTKIEASMALVDAKPIIELAPVDQTAYIREKDERCKTWMCRVRDE
D7F1G7_9VIRU_BATV     CSFKIVSNIDTVCSIEGPCTTFHNRLMITSTKQDYGIKMSCKTK-PGLTEEFQICKKTYTVLFATVEKNDKIEISTGDQTSFIQERDDRCKTWLCRVRDE
Q6PN63_9VIRU_NRIV     CSFKIASNIDTVCSIEGPCTTFHNRLMITSTKQDYGIKMSCKTK-PKETEEFQICKKTYTVLFTTVEKNDKIEISTGDQTSFIQERDDRCKTWLCRVRDE
GP_BUNYW_BUNV         CNFQIVSNIDTICSLEGPCDTFHNRISIKAMQQNYAVKLSCQKD-PRPSGTFKICNREYTVVFHTVAKDDKIEINVGDQTSFIKEKDDRCKTWLCRVRDE
A0A0R7FK51_9VIRU_UMB  CEIKVKTEIEASCKVEPPCPSYTNRIIIKPGRDTHTLLMKCDKKITSGELTIKICNMQIQAHLSTIESNDQLELSTGDQSTYIHEEDLRCSTWICKVRDE
```

```
A0A0R7FK55_9VIRU_WIT   CEANLKTTVETSCEVTSICSLYTNRLILLPSQEKYSLMIRCDRQIAENELDISICGKKIESHITITNTNDKLELSTGEQSTYIHEEDLRCTTWLCKFQEE
A0A2I4SI68_9VIRU_TCM   CRLEIPTSTQSTCTIKSNCETFINRIVISPNDKNYHLKFQCRTK--TDYVSLDICGNKKDISLKLSSVKPQLEVLPQAESAYVKERDDKCSTWLCKVKDQ
Consensus              C     t     C ! s C  % #ri i p    y lk  C  k        i ICg  i            kl#    d#t %!kE D rC TWlCrv ##
Prim.cons.             CE2KI3TNIETSC2IESPCTTFHNRI3ITP33Q2YALKMSC32KIP22TLT2KICNKKITV32TTVEKNDKLELSTGDQTSYIKE2DDRCKTWLCRVRDE

                             1410      1420      1430      1440      1450      1460      1470      1480
                                |         |         |         |         |         |         |         |
Q806D6_9VIRU_AKAV      GIGFVLEPLWNELSLWGKYVLLALAIIISVLILVKVIRPLARYVVAVLKENDKVYKLENKLK--------------------------
A0A1I9WAL5_9VIRU_CEV   GIQVILEPFKNLFGSYIGIFYTSILIVITIFIIIYVILPICFKLKDTLQKHEDAYKREMKIR--------------------------
F6JSE3_BUNLC_LACV      GLQVILEPFKNLFGSYIGIFYTFIISIVTLLVIIYILLPICFKLRDTLRKHEDAYKREMKIR--------------------------
D7F1G7_9VIRU_BATV      GISVIFEPIRAFFGSYFSIAFYIVIGIIVLFLVIYIFLPMMFKLRDVLKRNEYLYLQEIKHK--------------------------
Q6PN63_9VIRU_NRIV      GISVIFEPIRAFFGSYFSIAFYVVISIIVLFLAIYIFLPMVFKLRDVLKRNEYLYLQEIKHK--------------------------
GP_BUNYW_BUNV          GISVIFEPIKAFFGSYFSIFFYIIVVVVVGFLIIYIFMPMFMKLKEVLKANEKLYLQEIKQK--------------------------
A0A0R7FK51_9VIRU_UMB   GTSFIFKPLTDWLGSFTKPILIAGCIIIVTIILIYILMPMCARLRDLLKKNEYEYLQDTKVIVKPKTAANSIGLRSKKINIQSSKAVD
A0A0R7FK55_9VIRU_WIT   GFNFILGPIYNWLGKFTWPVIAIIIIFLIFIGVYVFMPMCMKLRDILKKNEYEHLQEIKTDMNNIPMIVKYKIKPFA----------
A0A2I4SI68_9VIRU_TCM   GIGFLFSPFTGFFGEVWHYVVLFLAVMITFFIVVYILIPCLFKCRDILRKQQAEFIVERKLR--------------------------
Consensus              Gi fileP  n fg     v     iii  fi !y!  P   klrd Lkkn#  y  # K
Prim.cons.             GISVIFEPIKNFFGSYFSI2FYIIIIIIVLFIIIYIFLPMCFKLRDVLKKNEY2YLQEIK3K2222222222222222INIQSSKAVD
```

Table S5: Sequence alignments of different *Phenuiviridae* M polyproteins. NSm is highlighted in grey. Red type/ asterisk (*), green type/colon (:), and blue type/dot (.) indicate identical amino acid residues, conserved substitution, and semi-conserved substitutions respectively.

```
                          10        20        30        40        50        60        70        80        90       100
                           |         |         |         |         |         |         |         |         |         |
GP_RVFV            -MYVLLTILISVLVCEAVIRVSL-----SSTREETCFGDSTNPEMIEGAWD---------SLREE-EMPEELSCS-------------------------
A0A143Q355_TOSV    -MFIAKLLLISCLVQQAYLKYTIISEIGNGTRTSFCYSSRTSLGSLLEAWDNITESIGLTSGTCKVGSLENRDCKRDIMSNYVAEVHSNLHGLGAMMHNY
I1SV55_9VIRU_AMTV  ----------------------------------------------------------------------------------------------------
GP_PTPV            MIFTILNVLTRAMLVMSMYSLTTWD---STSRNDMCFSNDSPLEGLVYYWET-------HSKRHDYKKQESQRCR-----------------------V

Prim.cons.         MMF33L33LIS3LV33A3333T322EIGS3TR333CFS33T3LE3L33AWD2ITESIGL2S3R332333E333C3RDIMSNYVAEVHSNLHGLGAMMHN2

                         110       120       130       140       150       160       170       180       190       200
                           |         |         |         |         |         |         |         |         |         |
GP_RVFV            -------------ISGIRE-----VKTSSQEL----------------YRALKAIIAAD-------GL--NNI--------TCHGK-----------DPE
A0A143Q355_TOSV    GDDKRVEVLNDVIPDELNPGRHNCSDLDRKPFWTEFIDVAKPTNKIAPMSVIPPPIVKPIPV--AESTIKKSIP-FDKLLSEAEKELNQT-KIRLETERR
I1SV55_9VIRU       --------------------------MHQITVVSGPTEVSTCFGSLHPFQSLKPVMANALGV--LEGKMFCSIG-GRSLEEDDEGVLAVV-QGLQDSQQE
GP_PTPV            GDSDKKMITNVTIISLISE-----IQKSISELSLSCVNDDNSTGQVLTFNGLEDTIRGDYIVDCVTGLYQSDIGVGVGLGRTHHGHQQMKNKAVVIDEKE
                                                  .                   :   :          .    .*          .             : .
Prim.cons.         GD2222222N22IIS3I3EGRHNC334S44EL333333V333TG333PF44LKP4IA4D33VDC3EGL334SIGVG33L33T42G4L333NK33333E4E

                         210       220       230       240       250       260       270       280       290       300
                           |         |         |         |         |         |         |         |         |         |
GP_RVFV            DKISLIKGPPH----------------KKRVGIVRCER---RRDAKQIGRETMAG------------------------------IAMTV--LPALAVFA
A0A143Q355_TOSV    SNAEEAAKERARMREIEDKLRKDWELERERKDAVADDLA--KEIARRREIERKAHELDKMLKKKEREDLVARERVSEDMRNHVRKPNTTIAPAPTIITTL
I1SV55_9VIRU       EEISCHGTPKGKLVKYKRHPRK----ASGKKGAVDCVSG--KELKPQKKRGDLAH--------------ISEHFQADEVRSSSVNISTTL---KTILVAA
GP_PTPV            RMISLLETQQSENDIKMQVLMSEIEQLKNQLSKKRNERGQEKRDAEKVMSDLMARNSDLRKHNDILTAEISQMKNKNTIQRNKNTVSTTV--VPAILSVA
                      .                         : .         :.   :      *                                  *:    ::
Prim.cons.         44ISL44TP4433333333LRK22E23K4RKGAVRCERGQEK2DA4Q44RE4MAH22D22222222222IS33333333R33333ISTTVAP3P2ILV4A

                         310       320       330       340       350       360       370       380       390       400
                           |         |         |         |         |         |         |         |         |         |
GP_RVFV            L-----APVVFAED--------PHLRNRPGKGHNYIDGMTQEDATCKPVTYAGACSSFDVLLEKGKFPLFQSYAHHRTLLEAVHDTIIAKADPPS-CDLQ
A0A143Q355_TOSV    LMA---SAVMAGPLEN---RETNHLLNRPGNGAYTLSDFAES--TCT-LAYGSECKSWEHQLDELSFPFFHSNLDKYSMLEAATETIPILNKSSAVCTIS
I1SV55_9VIRU       LLT---SALVNG---------DPHIDNRPGKGDYA-RDASRSGNNCSPLKYGAACKAWTYQKDVSTYPFFNTFPYKYSLIEALSEGFIQMDEQNT-CKLT
GP_PTPV            LLSSSVAPIIAAPPDSPMINPWPHAKNRVGTGMYKYD--ENDDSGCRPIRYGVSCIGFDFMLKMDKYPFFNAFIGHKTPLESFADKIIEK-EEET-CEIG
                   *     :.:: .           *  ** *.*        ..   *  : *.  * .:    .  .:*:*::   : : :*:  : :    .  : * :
Prim.cons.         LL3SSV22VVA2P322PMI223PHL4NRPGKG4Y43DD344SD3TC4PL4YG4ACKS2D4QLD44K2PFFNSF442Y2LLEA442TII4K3E44TVC424

                         410       420       430       440       450       460       470       480       490       500
                           |         |         |         |         |         |         |         |         |         |
GP_RVFV            SA---HGNPCMKEKLVMKTHCPNDYQSAHYLNNDGKMASVKCPPKYGLTEDCNFCRQM---TGASL--KKGSYPLQDLFCQSSEDDGSKLKTKMKGVCEV
A0A143Q355_TOSV    PSTH-SSNACGREASLIKKKCGSNMSAFFYVNLAGQITVVKCDTNHVLSNDCGNCISK-TLSGQ-----KIYTPVQDMFCQKGWTESIPNTRYSKDLCSI
I1SV55_9VIRU       TTSPVTSESCLKSLRGIKHTCPPGFRSALFAHSTGKVRGIICDNQYQVTPDCKFCQST-ENNAE----VKNEIVIQDIVCQQNGTEYNGPIIKIPGYCGI
GP_PTPV            TN---KEFKCFEERAYIKGTCPTNINAVHYIDNKGKLRYVKCKENLEMTEDCAFCRKIKKKAGQSVQVQKTSVPLQDAICQENSDTYSGPKIPFKGVCKI
                   .        * ..   :*  *  .  :  : .  *::  : *  :  :: **  * .     .      *    :** .**..            . * :
Prim.cons.         T422V4SN4C4KE444IK4TCP4N442AHY4NN4GK4R4VKCD4NY4LTEDC4FCRS4K334GQS2QV3K4S4PLQD4FCQ4N42EYSGPKIK4KGVC4I

                         510       520       530       540       550       560       570       580       590       600
                           |         |         |         |         |         |         |         |         |         |
GP_RVFV            GVQAHKKCDGQLSTAHEVVPFAVFKNSKKVYLDKLDLKTEENLLPDSFVCFEHKGQYKGTMDSGQTKRELKSFDISQCPKIGGHGSKKCTGDAAFCSAYE
A0A143Q355_TOSV    GLHTVKECKTG-TTNFERVGFIVVEGR-KMYIEQMKMRSRQEFSEDQFLCYKSE-SSSG------SSVKLKKVKVESCKGVTTSSASKCSGDEYFCSRYP
I1SV55_9VIRU       EDVKYRDCSDK-DVSFEKVAFALMKNK-KLYLPSMVLKYVDTVSADHFRCFRHK-KAHGVTGSNTDATDYVRVKPRECKQLDSSKTTKCTGDEVFCSYYN
GP_PTPV            GLIKYKECKFK-TSSYETVSFITLKEKGKIYIEHLMLKNIEVVTNVSFVCYEHVGQDEQEVE----HRALKRVSVNDCKIVDNSKQKICTGDHVFCEKYD
                        :.*.      .* * *  .:   *:*:  : ::  : .    * *:.   .                ..  .*  :     . *:**  **. *
Prim.cons.         GL4KYKECK4KLTTSFE4V4F2V4KNK2K4Y2E424LK44E4VS4DSFVC2EHKGQ44G333S2234R4LKRVKV44CK4VD4SK4KKCTGDEVFCS4Y4

                         610       620       630       640       650       660       670       680       690       700
                           |         |         |         |         |         |         |         |         |         |
GP_RVFV            CTAQYANAYCSHANGSGIVQIQVSGVWKKPLCVGYERVVVKRELSAKPIQRVEPCTTCITKCEPHGLVVRSTGFKISSAVACASGVCVTGSQSPSTEITL
A0A143Q355_TOSV    CETANVEAHCILRRHSAVIEVNVNGVWIVPRCIGYEEVLVRRTSLKVEDTSSRECDTCLWECGKNKLIVKTHGPKIVYATACSHGSCKSVMQKPATFVYL
I1SV55_9VIRU       CDKERSDAFCFHANGSGIIQVQYGGSWLQPSCVGFETVMVTKPRLSLPLSANLNCPSCVAKCHQNSLEINSNGFLITSAIACSHGECKTKTQKPRHSIIM
GP_PTPV            CSTSYPDVTCIHAPGSGPLYINLMGSWIKPQCVGYERVLVDREVKQPLLAPEQNCDTCVSECLDEGVHIKSTGFEITSAVACSHGSCISAHQEPSTSVIV
                   *     :. *     *. : ::  * *  * *:*:* *:* :            * :*: :*  . : :.: *  *  * **: * * :  *.*   : :
Prim.cons.         C4T4Y4DA4CIHANGSGIIQ22V4G2WIKP4CVGYERVLV4RE4L44PL4444NCDTCV42C44NGL42KSTGFKITSAVACSHGSCK244QKPSTS2IL

                         710       720       730       740       750       760       770       780       790       800
                           |         |         |         |         |         |         |         |         |         |
GP_RVFV            KYPGISQSSGGDIGVHMAHDDQSVSSKIVAHCPPQDPCLVHGCIVCAHGLINYQCHTALSAFVVVFVFSSIAIICLAVLYRVLKCLKIAP---RK----V
A0A143Q355_TOSV    PYPGNSEIVGGDIGVHMTEESSPSNIHMVAHCPAKDSCEVSSCLFCVHGLLNYQCHTLFS----ALLISTTVMSILTLLLLVKGAKDLV---KRLFYWL
I1SV55_9VIRU       QKPSSLKISGGEVGVHLSTEGEEPSYKLSIHCDPINQCDAYSCTFCWENVLNFHCHTLISSFVLALMIGSVASCALAIMVKISKGTKSVILKSRNPVLWV
GP_PTPV            PYPGLLASVGGRIGIHLSHTSDSASVHMVVVCPPRDSCAAHNCLLCYHGILNYQCHSTLSAILTSFLLILFIYTVFSVTTNILYVLRLIP---KQ----L
                     *.     ** :*:*::  ..  . ::   * . : * . .* .* ..::*::**: :*     :::       :::   :    :      :.    :
Prim.cons.         PYPG42422GGDIGVH2SHES4S4S42MVAHCPP4DSC42HSCLFC4HGLLNYQCHTLLSAFV3A2LISS4A444LAVL44ILKGLK44PLKS24222W2
```

```
                         810       820       830       840       850       860       870       880       890       900
                           |         |         |         |         |         |         |         |         |         |
GP_RVFV           LNPLMWITAFIRWIYKKMVARVAHNINQVNREIGWMEGGQLVLGN-----PAPIPRH-A----PIPRYSTYL-ML-LLIVSYASACSELIQASSRITTCS
A0A143Q355_TOSV   ITPLCWLSVFCGWVIKSWKKRVGSAISRTNDTIGWR--DNRRYRQ-----DVERAQYTGGA--PGAKYSFYG-VMVLGLLGNVHSCSESIIADSKIMQCT
I1SV55_9VIRU      IKLVKWLWL---QIWKALNKGMTTLSRKINDQTDLEVGSNQSFKNGIPLREVVVQKRTGGTRIKPVNYYLYGSTIVLGLITGSFSCTENLIASSKISSCF
GP_PTPV           KSPVGWLKLFINWLLTALRIKTRNVMRRINQRIGWVDHHDV-----------ERPRHRE----PMRRFKTTL-LLTLIMMTGGNACSNTVVANSKQTRCV
                   . : *:      : .           : *   .     :               :          .:      : * ::    :*:: : *.*:   *
Prim.cons.        I4P24WL4LFI3WI4KAL4KRV444IRRIND4IGW43G4N4333NGIPLR3VERPRHTGG2RIP44RYSTY2S4LVLGL4TG442CSE4IIASSKIT4C4

                         910       920       930       940       950       960       970       980       990      1000
                           |         |         |         |         |         |         |         |         |         |
GP_RVFV           TEGVNTKCRLSGTALIRAGSVGAEACLMLKGVKEDQTKFLKIKTVSSELSCREGQSYWTGSISPKCLSSRRCHLVGECHVNRCLSWRDNETSAEFSFVGE
A0A143Q355_TOSV   TSGSSTLCKASGTVIMKLGPIGSESCLILKGLKDSEKQFISIKTISSELTCREGESFWTTLYTPVCLSSRRCHLMGECVSDRCLKWKTNETSAEFTGKAH
I1SV55_9VIRU      IESGRHVCKLSGVVNLRVGTIGSESCLIVKGPLDGQTQALKIRTKSSELVCQEGQSFWTNHFTPKCYSSRRCHLVGECQASNCLEWNASTVSQEFRSQYE
GP_PTPV           QEGSNTKCSITATITLRAGVIGAESCFIIKGPMENQQKTISIKTISSETVCREGSSFWTSLYIPSCLSSRRCHLVGDCVGNKCQSWRDDQLSREFSGVKD
                   ..    *  :..  :: * :*:*:*:::**  :.: : :.*:* ***  *:**.*:**    * * *******:*:*  ..* .*. .  * **    .
Prim.cons.        TEGSNTKCKLSGTV4LRAG4IG2ESCLILKGPK24QT2F22IKTISSELVCREGQSFWT4LYTPKCLSSRRCHLVGECV4NRCLSWRDNETSAEFSGV4E

                        1010      1020      1030      1040      1050      1060      1070      1080      1090      1100
                           |         |         |         |         |         |         |         |         |         |
GP_RVFV           STTMRENKCFEQCGGWGCGCFNVNPSCLFVHTYLQSVRKEALRVFNCIDWVHKLTLEITDFDGSVSTIDLGASSSRFTNWGSVSLSLDAEGISGSNSFSF
A0A143Q355_TOSV   GEVVHENRCFEQSGGIGYGCFNVNPSCLYVHSYLKSVYKNGFKIFRCVAWNHRIRLEVTTHSRKFPMV-LMAMSTQPTDWGSIGLILDSEGITGTNAYSF
I1SV55_9VIRU      PGMIVENVCFEQCGGIGCSCFNVNPSCLFGMTILKPTYDKAIKVFQCASWTHRITLEFEGPGITAKQVSLTSMSTQVAEWGSITLSVDADSTIDNPNISF
GP_PTPV           NHIMNENKCFEQCGAIGCGCFNINPSCLYVHAYLKSARNEAVRVFSCSDWVHRVSFEVKGPDGETELVTLGSPGTKFLNWGTLSLSLDAEGISGTNSISF
                     : ** ****.*. * .***:*****:  : *:.. .:..::* *  * *:: :*.   .     : * : .::  :**:: * :*::. ..    **
Prim.cons.        444M4ENKCFEQCGGIGCGCFNVNPSCL2VHTYLKSV2KEA42VF4C4DWVHRITLEVTGPDG4444V3LG2MSTQFTNWGSISLSLDAEGISGTNSISF

                        1110      1120      1130      1140      1150      1160      1170      1180      1190      1200
                           |         |         |         |         |         |         |         |         |         |
GP_RVFV           IESPSKGYAIVDEPFSEIPRQGFLGEIRCNSESSVLSAHESCLRAPNLISYKPMIDQLECTTNLIDPFVVFERGSLPQTRNDKTFAASKGNRGVQAFSKG
A0A143Q355_TOSV   MKHGSGSFAIIDDPYSPEPRKGFLGEVRCPTEETAIRASPSCKMAPNLIEYQPEMDTAECTTNMIDPMAIFNRGSLPQVRDGMTFTQSIEKNTVQALTTG
I1SV55_9VIRU      LKTREGEYALIEESITTSPQKGRLGEIRCQTEQQAAIGSPSCLQADKLVDYRPQYDTLECISKLIDPRAILKRNGLPQSRGKYLYTPSLGSDTVQAVSRN
GP_PTPV           LESSKGGFALYDEGYNEIPREGFLGEIRCSSESAAISAHKSCIRAPGLIKYKPMTDQIECTASLVDPFAIFLKGSLPQTRNGQTFTSTKDKKTVQAFTNG
                  ::  .  :*: ::  .  *::* ***:** :*.  .  .  **  *  *:.*:*  *  ** :.::** .:: :..*** *.  :: :  .  ***.: .
Prim.cons.        L2S4SGG2A2IDEPYSEIPRKGFLGEIRC42ES4AISA2PSCLRAPNLI4YKPM4D2LECTTNLIDPFAIF4RGSLPQTRNG4TFT4SKGK4TVQAF24G

                        1210      1220      1230      1240      1250      1260      1270      1280      1290      1300
                           |         |         |         |         |         |         |         |         |         |
GP_RVFV           SVQADLTLMFDNFEVDFVGAAVSCDAAFLNLTGCYSCNAGARVCLSITSTGTGSLSAHNKDGSLHIVLPSENGTKDQCQILHFTVPEVEEEFMYSCDGDE
A0A143Q355_TOSV   EVRASIRLTLDDYDVVYQNSQSDCSATFKNLTGCYSCDEGSRMCYQVKAEGETIFHFINEDESINVINKVNPDVSDYCTVLHFSRPVISIEGRYDCGLSK
I1SV55_9VIRU      TIEATMTLTLDNYEVSFLSEKTECLASFVNISGCYSCHSGASICFKVTSKTPASIYIRDEKETKIISKSITAGEKVICSVLHYSKPIVDEIFEYTCGSEW
GP_PTPV           AIKALLSINLDDHEIVFINKVKNCDATFLNVSGCYSCDYGAHVCVKVKSSESADFFAESEDKTTVLSFPIQSGTHDYCQVLHFQKPLVDERLSYSCGSEP
                   :.* : : :*:.:: : .   .* *:* *::*****. *: :* .:.:     :   .:. :  :      .    * :**:  * :.    * *. .
Prim.cons.        424A4LTLTLD2YEVVF4N4444CDATFLNL2GCYSCD4GARVC4KV2S4G4ASF4A4NEDE244IS4PI44GTKDYCQVLHFSKP4VDEEF4YSCGSE4

                        1310      1320      1330      1340      1350      1360      1370      1380      1390      1400
                           |         |         |         |         |         |         |         |         |         |
GP_RVFV           RPLLVKGTLIAIDPFDDRREAGGESTVVNPKSGSWNFFDWFSGLMSWFGGPLKLYSSFACMLHYQLGSFSSLYILEEQASLKCGLLPLRRPHRSVRVKVI
A0A143Q355_TOSV   RPMVIKGTLIATAPHDDRKHEGGSSLIVNPKGGTVDFFGWVSGLTSWLGGPLKTFLTILGFLALGLLLVVIIIII------------ARTGVQQALKKKN
I1SV55_9VIRU      KKIQVHGSLILLSLIDDHK-EGGSTIVVGPSSSSFSVGDWTTGLLSWFGGPIKTLTLLIIYVVATIIVIALVVAL------------IKLALKTMILKKN
GP_PTPV           KLIVIKGTLVCMGVYDFRNKTGGSSTVVNPSEGAWSISNWFSGLLDWLGGPMKAILKILGFIAIGIVCFVLFMIL------------IRIAVNSINIKKK
                  : : ::*:*:     * :.  **.: :*.*. .: .. .* :** .*:***:*    :   :   :  .  .  :             :   .    *
Prim.cons.        2PIV2KGTLIA44P4DDRK3EGGSSTVVNP2SGSWSFFDWFSGLLSW2GGPLKT4L4ILGFLA4G244FVL44ILEEQASLKCGLLPIR4AV4S444KKN


GP_RVFV           C
A0A143Q355_TOSV   K
I1SV55_9VIRU      V
GP_PTPV           N

Prim.cons.        4
```

Table S6: Sequence alignment of *Phenuiviridae* NSm. Red type/ asterisk (*), green type/colon (:), and blue type/dot (.) indicate identical amino acid residues, conserved substitution, and semi-conserved substitutions respectively.

```
                          10        20        30        40        50        60        70        80        90       100
                           |         |         |         |         |         |         |         |         |         |
GP_RVFV           MY--VLLTILISVLVCEAVIRVSLSS-----TREETCFGDSTNPEMIEGAWDSLREE---EMPEELSCSISGIREVKTSSQELYRALKAIIAAD--GLNN
A0A143Q355_TOSV   MF--IAKLLLISCLVQQAYLKYTIISEIGNGTRTSFCYSSRTSLGSLLEAWDNITES---IGLTSGTCKV-GSLENRDCKRDIMSNYVAEVHSNLHGLGA
I1SV55_9VIRU      ------------------MHQITVVSG---PTEVSTCFGSLHPFQSLKPVMANALGV----LEGKMFCSI-GGRSLEEDDEGVLAVVQGLQDSQ--QEEE
GP_PTPV           MIFTILNVLTRAMLVMSMYSLTTWDST----SRNDMCFSNDSPLEGLVYYWETHSKRHDYKKQESQRCRV-GDSDKKMITNVTIISLISEIQKSISELSL
                                        :  *     :. . *:..      :     .           .  * : *  . .   .       .    .
Prim.cons.        M3FTIL33LLIS3LV33AY444T44S3IGN2TR4STCF2S4TPLESL44AWDN44E4HDY344ES44CS2SG4RE4K444444444L4AEI4S422GL44

                         110       120       130       140       150       160       170       180
                           |         |         |         |         |         |         |         |
GP_RVFV           ITCHGKDPEDKISLIKGP-PHKKRVGIVRCERRRDAKQIGRETMAGIAMTVLPALAVFALAPVVFA----------------
A0A143Q355_TOSV   MM-HNYGDDKRVEVLNDVIPDELNPGRHNCS-DLDRKPFWTEFID-------------------------------------
I1SV55_9VIRU      ISCHGTPKGKLVKYKRHPRKASGKKGAVDCVSGKELKPQKKRGDLAHISEHFQADEVRSSSVNISTTLKTILVAALLTSALVN
GP_PTPV           SCVNDDNSTGQVLTFNGLEDTIRGDYIVDCVTGLYQSDIGVGVGLGRTHHGH------------------------------
                     :.      :   .             *      .
Prim.cons.        I4CHG4444K4V444NGP3P44444GIVDCV3GLD4KPIG4E44LG3333332A22V222222222TLKTILVAALLTSALVN
```

Table S7: Sequence alignment of *Phenuiviridae* Gn^{cyto}. Red type/ asterisk (*), green type/colon (:), and blue type/dot (.) indicate identical amino acid residues, conserved substitution, and semi-conserved substitutions respectively.

```
                          10        20        30        40        50        60        70        80        90       100
                           |         |         |         |         |         |         |         |         |         |
GP_RVFV           AVLYRVLKCLKIAPRKVLNPLMWITAFIRWIYKKMVARVAHNINQVNREIGWMEGGQLVLGNPAPIPRH-APIPRY-------STYLMLLLIVSYASA--
A0A143Q355_TOSV   LLVKGAKDLVKRLFYWLITPLCWLSVFCGWVIKSWKKRVGSAISRTNDTIGWRDNRRYRQDVERAQYTGGAPGAKY-------SFYGVMVLGLLGNVHS-
I1SV55_9VIRU      AIMVKISKGTKSVILKSRNPVLWVIKLVKWLWLQIWKALNKGMTTLSRKIN--DQTDLEVGSN-QSFKNGIPLREVVVQKRTGGTRIKPVNYYLYGSTIV
GP_PTPV           SVTTNILYVLRLIPKQLKSPVGWLKLFINWLLTALRIKTRNVMRRINQRIGWVDHHDV------ERPRHREPMRRF-------KTTLLLTLIMMTGGN--
                   :        :       .*: *:  :  *:           :   .  *.  :                 *  .

Prim.cons.        AV444ILK4LK44P4KL4NP24WL44FI4WL4K444KRV44424R4NR4IGW3D44DL33G33244PRHGAP4RRYVVQKRTGSTYL4LVLI4LYGS42V


GP_RVFV           ----
A0A143Q355_TOSV   ----
I1SV55_9VIRU      LGLI
GP_PTPV           ----

Prim.cons.        LGLI
```

Table S8: Sequence alignment of RVFV Gn^{cyto} and NSm. Red type/ asterisk (*), green type/colon (:), and blue type/dot (.) indicate identical amino acid residues, conserved substitution, and semi-conserved substitutions respectively.

```
                       10        20        30        40        50        60        70        80        90       100
                        |         |         |         |         |         |         |         |         |         |
GP_RVFV_Gncyto-------------------------------------AVLYRVLKCLK--IAPRKV---LNPLMWITAFIRWIYKKMVARVAHN-INQV-------NREI
GP_RVFV_NSm   MYVLLTILISVLVCEAVIRVSLSSTREETCFGDSTNPEMIEGAWDSLREEEMPEELSCSISGIREVKTSSQELYRALKAIIAADGLNNITCHGKDPEDKI
                                                    ::  . ..*:    *.::   :. :  :.:  : :*: : * :* : :*::       : :*
Prim.cons.   MYVLLTILISVLVCEAVIRVSLSSTREETCFGDSTNP222222222L2EE22P222SCS22222222222222Y2222A22A22G2N22TCHGKDP222I

                      110       120       130       140       150
                        |         |         |         |         |
GP_RVFV       GWMEG--GQLVLG--------NPAPIPRHAPIPRYSTYLMLLLIVSYASA---
GP_RVFV       SLIKGPPHKKRVGIVRCERRRDAKQIGRETMAGIAMTVLPALAVFALAPVVFA
              . ::*   :  :*        :.  * *.:      * *  * :.: *..
Prim.cons.    2222GPP22222GIVRCERRR2222I2R22222222T2L22L22222A22VFA
```

Table S9: Sequence alignment of TSWV G1/2 and NSm. Red type/ asterisk (*), green type/colon (:), and blue type/dot (.) indicate identical amino acid residues, conserved substitution, and semi-conserved substitutions respectively. Identity is 6 %.

```
                              10        20        30        40        50        60        70        80        90       100
                               |         |         |         |         |         |         |         |         |         |
TSWV_S480911_NSm      ----------------------------------------------------------------------------------------------------
TSWV__S480911_G12     MRILKLLELVVKVSLFTIALSSVLLAFLIFRATDAKVEIIRGDHPEIYDDSAENEVPTAASIQREAILETLTNLMLESRTPGTRQIREEKSTIPISAEPT

Prim.cons.            MRILKLLELVVKVSLFTIALSSVLLAFLIFRATDAKVEIIRGDHPEIYDDSAENEVPTAASIQREAILETLTNLMLESRTPGTRQIREEKSTIPISAEPT

                             110       120       130       140       150       160       170       180       190       200
                               |         |         |         |         |         |         |         |         |         |
TSWV_S480911_NSm      ----------------------------------------------------------------------------------------------------
TSWV__S480911_G12     TQKTISVLDLPNNCLNASSLKCEIKGISTYNVYYQVENNGVIYSCVSDSAEGLEKCDNSLNLPKRFSKVPVIPITKLDKKRHFSVGGKFFISESLTQDNY

Prim.cons.            TQKTISVLDLPNNCLNASSLKCEIKGISTYNVYYQVENNGVIYSCVSDSAEGLEKCDNSLNLPKRFSKVPVIPITKLDKKRHFSVGGKFFISESLTQDNY

                             210       220       230       240       250       260       270       280       290       300
                               |         |         |         |         |         |         |         |         |         |
TSWV_S480911_NSm      ----------------------------------------------------------------------------------------------------
TSWV__S480911_G12     PITYNSYPTNGTVSLQTVKLSGDCKITKSNFANPYTVSITSPEKIMGYLIKKPGENVEHKVISFSGSASITFTEEMLDGEHNLLCGDKSAKIPKTNKRVR

Prim.cons.            PITYNSYPTNGTVSLQTVKLSGDCKITKSNFANPYTVSITSPEKIMGYLIKKPGENVEHKVISFSGSASITFTEEMLDGEHNLLCGDKSAKIPKTNKRVR

                             310       320       330       340       350       360       370       380       390       400
                               |         |         |         |         |         |         |         |         |         |
TSWV_S480911_NSm      ----------------------------------------------------------------------------------------------------
TSWV__S480911_G12     DCIIKYSKSIYKQTACINFSWIRLILIALLIYFPIRWLVNKTTKPLFLWYDLMGLITYPVLLLINCLWKYFPLKCSNCGNLCIVTHECTKVCICNKSKAS

Prim.cons.            DCIIKYSKSIYKQTACINFSWIRLILIALLIYFPIRWLVNKTTKPLFLWYDLMGLITYPVLLLINCLWKYFPLKCSNCGNLCIVTHECTKVCICNKSKAS

                             410       420       430       440       450       460       470       480       490       500
                               |         |         |         |         |         |         |         |         |         |
TSWV_S480911_NSm      ----------------------------------------------------------------------------------------------------
TSWV__S480911_G12     KEHSSECPILSKEADHDYNKHKWTSMEWFHLIVNTKLSLSLLKFVTEILIGLVILSQMPMSMAQTTQCLSGCFYVPGCPFLVTSKFEKCSEKDQCYCNVK

Prim.cons.            KEHSSECPILSKEADHDYNKHKWTSMEWFHLIVNTKLSLSLLKFVTEILIGLVILSQMPMSMAQTTQCLSGCFYVPGCPFLVTSKFEKCSEKDQCYCNVK

                             510       520       530       540       550       560       570       580       590       600
                               |         |         |         |         |         |         |         |         |         |
TSWV_S480911_NSm      ----------------------------------------------------------------------------------------------------
TSWV__S480911_G12     EDKIIESIFGTNIVIEGPNDCIENQNCIARPSIDNLIKCRLGCEYLDLFRNKPLYNGFSDYTGSSLGLTSVGLYEAKRLRNGIIDSYNRQGKISGMVAGD

Prim.cons.            EDKIIESIFGTNIVIEGPNDCIENQNCIARPSIDNLIKCRLGCEYLDLFRNKPLYNGFSDYTGSSLGLTSVGLYEAKRLRNGIIDSYNRQGKISGMVAGD

                             610       620       630       640       650       660       670       680       690       700
                               |         |         |         |         |         |         |         |         |         |
TSWV_S480911_NSm      ----------------------------------------------------------------------------------------------------
```

```
TSWV__S480911_G12       SLNKNETSIPENILPRQSLIFDSVVDGKYRYMIEQSLLGGGGTIFMLNDKTSETAKKFVIYIKSVGIHYEVSEKYTTAPIQSTHTDFYSTCTGNCDTCRK

Prim.cons.              SLNKNETSIPENILPRQSLIFDSVVDGKYRYMIEQSLLGGGGTIFMLNDKTSETAKKFVIYIKSVGIHYEVSEKYTTAPIQSTHTDFYSTCTGNCDTCRK

                               710       720       730       740       750       760       770       780       790       800
                                 |         |         |         |         |         |         |         |         |         |
TSWV_S480911_NSm        ------------------------------------------MDAS-------KGKILLNIEGTSSFGTYESDSITESEGYD---LSARMIVD-TNHHIS
TSWV__S480911_G12       NQALTGFQDFCVTPTSYWGCEEAWCFAINEGATCGFCRNIYDMDKSYRIYSVLKSTIVADVCISGILGG-QCSRITEEVPYENTLFQADIQADLHNDGIT
                                                                  ** *       *..*: ::  :. :*  :.. ***.  *:   :.* : .*  *. *:
Prim.cons.              NQALTGFQDFCVTPTSYWGCEEAWCFAINEGATCGFCRNIYDMD2SYRIYSVLK22I2222222222G2Y2222ITE222Y2NTL22A2222DL2N22I2

                                                                                  NSm   100
                                                                                          |
                               810       820       830       840       850       860       870       880       890       900
                                 |         |         |         |         |         |         |         |         |         |
TSWV_S480911_NSm        -------NWKNDLFVGNGKQNANKVIKIC--PTWDSRKQYMMISRIV-----IWVCPTIPNPTGKLVVALIDP-NMPSGKQVILKGQGTITDPICFVFYL
TSWV__S480911_G12       IGELIAHGPDSHIYSGN-IANLNDPVKMFGHPQLTHDGVPIFTKKTLEGDDMSWDCAAIGKKSVTIKTCGYDTYRFRSGLEQISDIPVSFKDFSSFFLAK
                               . ...:: **   * *. :*:   *        :: .: :      * *.:* : : .: ..  *. .: ** : * .   ::.*  .*.:
Prim.cons.              IGELIAH22222222GNG22N2N222K22GHP222222222222222EGDDM2W2C22I222222222222D2Y222SG222I2222222D222F2222

                               910       920       930       940       950       960       970       980       990      1000
                                 |         |         |         |         |         |         |         |         |         |
TSWV_S480911_NSm        NWSI--------------------PKMNNTPENC-----CQLHLMCSQEYKKGVSFGSVM---------YSWTKEFGDSPRADKDKSCMVIPLNRAIRAR
TSWV__S480911_G12       SFSLGKLKMVVDLPSDLFKVAPKKPSITSTSLNCNGCLLCGQGLSCLLEFFSDLTFSTAISIDACSLSTYQLAVKKG-SNKYNITMFCSANPDKK----K
                        .:*:                    *.:..*. **     *   * *  *: ..::*.:.:         *. : : * * : :    * . * ::    :
Prim.cons.              22S2GKLKMVVDLPSDLFKVAPKKP2222T22NCNGCLLC222L2C22E222222F2222SIDACSLSTY222222GDS22222222C222P222AIRA2

                                                         NSm   240
                                                                 |
                              1010      1020      1030      1040      1050      1060      1070      1080      1090      1100
                                 |         |         |         |         |         |         |         |         |         |
TSWV_S480911_NSm        SQAFIEACKLIIPKGNSEKQIKKQLKELSSNLERSVEEEEEGISDSVAQLSFDEI---------------------------------------------
TSWV__S480911_G12       MTLYPEGNPDISVEVLVNNVIVEEPENIIDQNDEYAHEEQQYNSDSSAWGFWDYIKSPFNFIASYFGSFFDTIRVVLLIAFIFLVTYFCSILTSICKGYV
                           : *.   *  :   :: * :: ::: .: :. ..**::  *** *   :* *
Prim.cons.              22222E2222I222222222I2222222222222222EE2222SDS2A2222D2IKSPFNFIASYFGSFFDTIRVVLLIAFIFLVTYFCSILTSICKGYV

                              1110      1120      1130      1140
                                 |         |         |         |
TSWV_S480911_NSm        ----------------------------------------
TSWV__S480911_G12       KNESYKSRSKIEDDDEPEIKAPMLMKDTMTRRRPPMDFSHLV

Prim.cons.              KNESYKSRSKIEDDDEPEIKAPMLMKDTMTRRRPPMDFSHLV
```

Table S10: Sequence alignment of different Tospovirus G1/2. Asterisk (*),colon (:), and dot (.) indicate identical amino acid residues, conserved substitution, and semi-conserved substitutions respectively. Alignment was done using Clustal from the Uniprot website. Uniprot P36291 is identical to Genebank S48091. Uniprot P89252 is Genebank U75379, for WSMV (*Watermelon silver mottle virus*), Uniprot Q84405 is Genebank U42555 for GBNV (*Groundnut bud necrosis virus*) and Uniprot Q01260 is M74904 for INSV (*Impatiens necrotic spot virus*).

```
tr|P89252|P89252_WASMV      MKKYYLL-------VYCLGLASLLFVPDVYLLNQVDDSVQLKRI---------------- 37
tr|Q84405|Q84405_9VIRU      MKKYYLL-------VYCLGLVSLFFVSEVYLLNQVDNSVQLRKI---------------- 37
sp|P36291|GP_TSWV1          MRILKLLELVVKVSLFTIALSSVLL--AFLIFRATDAKVEIIRGDHPEIYDDSAENEVPT 58
sp|Q01260|GP_INSV           -----------------------------MALKETDAKIHVERGDHPEIYDEAYYDRSVD 31
                                                           :. .* .:.: :

tr|P89252|P89252_WASMV      ----QDRYRVDDPEDLIEKEEPTTIRVTRLKEEKLSR----ILRNDGSTTQS--P--VLE 85
tr|Q84405|Q84405_9VIRU      ----QDRYKVDDPEDLVEEEDNIITEIIEPRQKKLSR----ILRNDEVTTQS--S--VSE 85
sp|P36291|GP_TSWV1          AASIQREAILETLTNLMLESRTPGTRQIREEKST-------IPISAEPTTQKTISVLDLP 111
sp|Q01260|GP_INSV           ----HKNEILDTLAEMLQNATGKTLRPTRDTQTVLANNEVPQSSSGLSSTPTTISIMDLP 87
                                : .  ::   ::: :      .  .  :            .   :* .

tr|P89252|P89252_WASMV      LSCDNFEKRHCVIKGVSEFNAHYQIDTGDKIISCINNSANIFDICQYEKEFKKIKFKNFP 145
tr|Q84405|Q84405_9VIRU      LSCDIFEKRHCMIKGVSDFNAHYQIDNGNEIISCISNSANIFDICQYEKEFKKIKFKSFP 145
sp|P36291|GP_TSWV1          NNCLNASSLKCEIKGISTYNVYYQVENNGVIYSCVSDSAEGLEKCDNSLNLP-KRFSKVP 170
sp|Q01260|GP_INSV           NPCLNASSLTCSIKGVSTFNVYYQVESNGVIYSCISDTITKLGNCEGSSELP-RSFETVP 146
                              *   ..  * ***:* :*.:**::... * **:.::   :  *: . ::    *...*

tr|P89252|P89252_WASMV      VVPVLKLENKRVLEVGTKFFFVDKSN---NPINIDPKVDLKSPTVARLSVRLSGDCKINQ 202
tr|Q84405|Q84405_9VIRU      VVPVLKLENKKVLEIGTKFFFVDKSN---NPINIDPKVNLKSPTVARLSVRLSGDCKINQ 202
sp|P36291|GP_TSWV1          VIPITKLDKKRHFSVGGKFFISESLTQDNYPITYN--SYPTNGTVSLQTVKLSGDCKITK 228
sp|Q01260|GP_INSV           VVPITKIDNKRKLSIGTKFYIIESLENYNYPIMYN--SRPTNGTVSLQSVKFSGDCKISK 204
                            *:*: *:::*: :.:* **:: :.      **  :     .. **:  :*::******.:

tr|P89252|P89252_WASMV      VSMSSPYQIKLRSEEN-IGLLIKDVKNSKPANIKSITGDSTINFKPEELDGNHFLLCGDK 261
tr|Q84405|Q84405_9VIRU      VSMSSPYQIKLRSEEN-IGILIKNVKSSKSGNIKSIAGDTTINFKPEELDGNHFLLCGDK 261
sp|P36291|GP_TSWV1          SNFANPYTVSITSPEKIMGYLIKKPGENVEHKVISFSGSASITFTEEMLDGEHNLLCGDK 288
sp|Q01260|GP_INSV           TNIVNSYTVSLTTPEKIMGYVVKREGSDMSHSIISFSGSVSLTFTEENMDGKHNLLCGDK 264
                             .: . * :.: : *: :* ::*   ..   .: *::*. ::.*. * :**:* ******

tr|P89252|P89252_WASMV      SSLIAKVDIPVRNCVSKYSEEPKKIFFCTNFSYFKWIFVFLMITFPVSWLIWKTKDALSI 321
tr|Q84405|Q84405_9VIRU      SSLIAKVDIPVRNCVSKYSDEPKKIFFCTNFSYFKWIFVFLMVAFPISWFIWKTKDALSI 321
sp|P36291|GP_TSWV1          SAKIPKTNKRVRDCIIKYSKSIYKQTACINFSWIRLILIALLIYFPIRWLVNKTTKPLFL 348
sp|Q01260|GP_INSV           SSKVPLVDKRVRDCIIKYSKNIYKQTACINFSWFRLIMIALIVYFPIRYLVNKTSKTLFY 324
                            *: :  .:  **:*: ***..  *   * ***::: *:: *:: **: ::: **..  *

tr|P89252|P89252_WASMV      WYDVMGIVTYPILWVINWSWPYFPLKCGICGCFSFLTHSCSEKCVCNQSKASRGHSEECY 381
tr|Q84405|Q84405_9VIRU      WYDVIGILTYPILWTLNWLWPYFPLKCRICGCFSFLTHSCTEKCVCNQDKASKDHTDECY 381
sp|P36291|GP_TSWV1          WYDLMGLITYPVLLLINCLWKYFPLKCSNCGNLCIVTHECTKVCICNKSKASKEHSSECP 408
sp|Q01260|GP_INSV           GYDLLGLITYPILLLINYLWSYFPLKCKVCGNLCLVTHECSKLCICNKNKASEEHSEECP 384
                             **::*::***:*  :*  * ******  ** :.::**.*:: *:**:.***. *:.**
```

```
tr|P89252|P89252_WASMV      LSTRDKFE---WRKLTLIQQFQFIINTKLSSNFLVFVTKMILASILISYIPSSIALKQNN 438
tr|Q84405|Q84405_9VIRU      LFTKDKEE---WRKLTLIQQFQFIINTKLSTNFLVFVTKMILASILISYIPSSIALKQPN 438
sp|P36291|GP_TSWV1          ILSKEADHDYNKHKWTSMEWFHLIVNTKLSLSLLKFVTEILIGLVILSQMPMSM--AQTT 466
sp|Q01260|GP_INSV           IITRTAEK-NKKYNWASIEWFHLIVNTKIGLSFLKAVTETLIGFLILSQMPMSM--AQTA 441
                            : ::   .      : : :: *::*:***:. .:*  **: ::. :::* :* *:   *

tr|P89252|P89252_WASMV      LCVEKCYYNLNLDSLTTDKFGTMGNGYETCECSIG-NVITETVYRSGVPMSRATSLNDCV 497
tr|Q84405|Q84405_9VIRU      ICVEKCYYNLNLDSLTTDKFGMTDNGYETCECSIG-NVITETVYRSGVPMSRATSLNDCV 497
sp|P36291|GP_TSWV1          QCLSGCFYVPGCPFLVTSKFEKCSE-KDQCYCNVKEDKIIESIFGTNIVI---EGPNDCI 522
sp|Q01260|GP_INSV           QCLDSCYYVPGCDRFVTNRYDKCPE-KDQCFCAIKENSIVESNFLTNVVT---EGPMDCI 497
                             *:. *:*  .    :.*.::    :  : * * :  : * *: : :.:      .  **:

tr|P89252|P89252_WASMV      LGSD-LCMVSNNQAQNLFACRNGCNSLESIKNIPNIKFNKFYKGHSFKGNLTSLKIANRL 556
tr|Q84405|Q84405_9VIRU      LGSD-LCMVSNNQAQNLFACRNGCNSLSSIKNIPDTKFNKLYKGQSFKGNLTSLKIANRL 556
sp|P36291|GP_TSWV1          ENQNCIARPS---IDNLIKCRLGCEYLDLFRNKPLYNGFSDYTGSSLGLTSVGLYEAKRL 579
sp|Q01260|GP_INSV           PYQECKGRITENALVTFVKCRFGCEYASIFQSKPLDNGFLEYSGDTLGLNAVNLHFMKRL 557
                              .:     :     .:. ** **:  . ::. *  :    *.* ::  . ..*   :**

tr|P89252|P89252_WASMV      REGYMDSFTESKILEEESAKEYKFYKSL-KADDVPPRNLMPRQSLVFSTEVDGKYRYLLE 615
tr|Q84405|Q84405_9VIRU      REGYMDSPTESKILEEESAKEYKFYKSL-KVDDIPPENLLPRQSLVFSTEVDGKYRYLLE 615
sp|P36291|GP_TSWV1          RNGIIDSYNRQGKISGMVA-----GDSLNKNETSIPENILPRQSLIFDSVVDGKYRYMIE 634
sp|Q01260|GP_INSV           RNGIIDFYNKTEK-YGYIS-----GDALKSNESDIPESIFPRKSLIFDSVIDGKYRYMIE 611
                            *:* :*  ..         :      .:* . :   *..::**:**:*.: :******::*

tr|P89252|P89252_WASMV      MDIKSNTGSIYLLNDDAMHSPMEFIIYVKSVGVEYDVRYKYSTAKIDTTVADYLVTCTGN 675
tr|Q84405|Q84405_9VIRU      MDIKSNTGSVYLLNDDATHSPMEFMIYIKSVGVEYDVRYKYSTAKIDTTVSDYLVTCTGK 675
sp|P36291|GP_TSWV1          QSLLGGGGTIFMLNDKTSETAKKFVIYIKSVGIHYEVSEKYTTAPIQSTHTDFYSTCTGN 694
sp|Q01260|GP_INSV           ESLLSGGGTVFSLNDKSSSTAQKFVVYIKKVRIQYDVSEQYTTAPIQSTHTDFFSTCTGK 671
                             .: .. *::: ***.:  :  :*::*:*.* :.*:*  :*:** *::* :*:  ****:

tr|P89252|P89252_WASMV      CADCRKQNPKVGSLDFCVTPTSWWGCEELGCLAINEGSICGHCTSIYDLSSLVNIYQVVE 735
tr|Q84405|Q84405_9VIRU      CADCIKQKPKVGVLDFCVTPTSWWGCEELGCLAINEGSICGHCTNIYDLSSLVNIYQVVE 735
sp|P36291|GP_TSWV1          CDTCRKNQALTGFQDFCVTPTSYWGCEEAWCFAINEGATCGFCRNIYDMDKSYRIYSVLK 754
sp|Q01260|GP_INSV           CSDCRKEQPITGYQDFCITPTSYWGCEEVWCLAINEGATCGFCRNVYDMDQSFRIYSVIK 731
                            *  * *::  .*  ***:****:*****  *:*****: **.* .:**:..  .**.*::

tr|P89252|P89252_WASMV      SHVTAEICIKSLDGYSCKRHSDRSPIQTDYYQLDMSVDLHNDYMSTDKLFAVTKQQKILT 795
tr|Q84405|Q84405_9VIRU      SHVTAEVCVKSLDGYSCKKHSDRSPIQTDYYQLDMSIDLHNDYMSTDKLFAVTKQQKILT 795
sp|P36291|GP_TSWV1          STIVADVCISGILGGQCSRITEEVPYENTLFQADIQADLHNDGITIGELIAHGPDSHIYS 814
sp|Q01260|GP_INSV           STIKSEVCISGFVGAKCFTVSEEVPSESGYFQADILADFHNDGLTIGQLIAHGPDSHVYA 791
                            * : :::*:..: * .*    ::. * :.  :* *:  *:***  ::  .:*:*   :.:: :

tr|P89252|P89252_WASMV      GNIADLGDFSGSSFGHPQITIDGIPLSVPSTLSQNEFTWSCSAVGDKKVNIRQCGLYTYS 855
tr|Q84405|Q84405_9VIRU      GNIADLGDFAGSSFGHPQITIDGIPLSVPATLSQNDFTWSCSAVGDKKVNIKQCGLYTYS 855
sp|P36291|GP_TSWV1          GNIANLNDP-VKMFGHPQLTHDGVPIFTKKTLEGDDMSWDCAAIGKKSVTIKTCGYDTYR 873
sp|Q01260|GP_INSV           GNIARLNNP-SKMFGHPQLSHQGDPIFSKKTLDTNDLSWDCSAIGKKTITIKSCGYDTYR 850
                            **** *.:    . *****:: :* *:     **. ::::*.*:*:*.*.:.*: **  **

tr|P89252|P89252_WASMV      AIYVLSPSKDISHLDESKNKLYMEKDFLVGKLKMVIDMPKEMFKKIPTKPVLSEAKMVCS 915
tr|Q84405|Q84405_9VIRU      AIYVLSPSKDVSHLDENSNKLYMEKDFLVGKLKMVVDMPKEMFKKIPTKPIISEAKMICS 915
sp|P36291|GP_TSWV1          FRSGLEQISDIPVSFKDFSSFFLAKSFSLGKLKMVVDLPSDLFKVAPKKPSITSTSLNCN 933
sp|Q01260|GP_INSV           FKTGLNQISDIPVQFTDQNSFYMEKIFSLGKLKIVLDLPSELFKTVPKKPILSSVSLSCK 910
                                *.  .*:      . ..::: * * :****:*:*:*.::**  *.** ::...: *.

tr|P89252|P89252_WASMV      GCSKCAIGIDCNITYTSDTTFSARLMMDTCSFKSDQIGTFLGPNEKSIKAYCYEEITDKS 975
tr|Q84405|Q84405_9VIRU      GCSQCAVGIDCNITYTSDTTFSARLMMDTCSFKSDQLGTFLGPNEKSIKAYCSEEITDKS 975
sp|P36291|GP_TSWV1          GCLLCGQGLSCLLEFFSDLTFSTAISIDACSLSTYQLAVKKGSNKYNITMFCSANPDKKK 993
sp|Q01260|GP_INSV           GCFLCSQGLRCAASFISDITFSARLTMKQCSLSTYQIAVKKGANKYNLTMFCTSNPEKQK 970
                            **  *. *: *    : ** ***: : :. **:.: *:..   * *: .:. :*  :  .:.

tr|P89252|P89252_WASMV      LKLIPEDQQELTVDIQVDEFIQVDRDTIIHFDDKSAHDENKHHSDTSISSLWDWIKAPFN 1035
tr|Q84405|Q84405_9VIRU      LKLIPEDQEELTVDIQVDEFTQVDQDTIIHFDDKSAHDENKHHSDTSISSLWDWIKAPFN 1035
sp|P36291|GP_TSWV1          MTLYPEGNPDISVEVLVNNVIVEEPENIIDQNDEYAHEEQQYNSDSSAWGFWDYIKSPFN 1053
sp|Q01260|GP_INSV           MIIEPEGDKSYSVEALVDSVAVLEPENIIDQNDQHAHEEQQYNSDTSVWSFWDYVKSPFN 1030
                            : : **.: . :*:  *:..   : :.**. :*: **:*::::**:*  .:**::*:***

tr|P89252|P89252_WASMV      WVASFFGSFFDLVRVILVIMAVCVGVYIVSSIFKLSRTYYVDKRRQKLEDAMESVESSIL 1095
tr|Q84405|Q84405_9VIRU      WIASFFGSFFDLVRIILVIVAACVGLYIVSSIFKLSRTYYVDKRRQKLEDAIESVESSVL 1095
sp|P36291|GP_TSWV1          FIASYFGSFFDTIRVVLLIAFIFLVTYFCSILTSICKGYVKNES-YKSRSKIEDDDEPEI 1112
sp|Q01260|GP_INSV           FIASHFGSFFDTVRVVLLILFVFALAYLCSIVATMCRGYVRNKS-YKTKY-IEDTNDYSL 1088
                            ::**.****** :*::*:*       *: * : .:.: *  ::   * .  :*. :.  :

tr|P89252|P89252_WASMV      LTNFTRTDQTRKRKSPPKGYDLCLDI         1121
tr|Q84405|Q84405_9VIRU      LANYTGIDQTRKRKSPPKGYDFSLDI         1121
sp|P36291|GP_TSWV1          KAPMLMKD-TMTRRRPPMDFSHLV--         1135
sp|Q01260|GP_INSV           VSTSSGKD-TITRRRPPLDFSGI---         1110
                             :     * * .*: ** .:.
```
